# Effects of different menthol administration routes on endurance performance and physiological responses in the heat: a network meta-analysis

**DOI:** 10.3389/fnut.2026.1833420

**Published:** 2026-05-18

**Authors:** Yongliang Zhu, Junyu Zhao, Jiayi Yao, Haozhe Wang, Xiaotong Yuan

**Affiliations:** 1 School of Physical Education, Shandong Sport University, Jinan, China; 2 School of Physical Education, China University of Mining and Technology, Xuzhou, China

**Keywords:** administration routes, endurance performance, heat stress, mean power output, menthol, network meta-analysis, physiological responses, randomized controlled trials

## Abstract

**Objective:**

This study employed a network meta-analysis (NMA) to systematically evaluate and compare the relative efficacy of three menthol administration routes—ingestion (ING), mouth rinsing (MR), and topical application (Top)—on exercise performance and physiological responses in the heat, aiming to explore potential task-oriented personalized supplementation strategies.

**Methods:**

Databases including PubMed, Embase, Web of Science, and CNKI were searched for randomized controlled trials (RCTs) investigating menthol interventions on exercise performance in hot environments (T≥25 °C), with a search deadline of March 2, 2026. Two reviewers independently performed literature screening, data extraction, and risk of bias assessment using the Cochrane RoB 2.0 tool. Network meta-analysis was conducted using Stata 18.0 software.

**Results:**

The network meta-analysis of the 15 included randomized controlled trials revealed outcome-specific trends in exercise performance, although most pairwise comparisons did not reach statistical significance. Surface under the cumulative ranking curve (SUCRA) analysis indicated that ingestion (ING) demonstrated a potential trend for improving endurance performance (SUCRA = 76.1%), while mouth rinsing (MR) showed a tendency for enhancing mean power output (SUCRA = 66.9%). Regarding physiological responses, the impact of all administration routes on core temperature (Tc) and heart rate (HR) was modest and lacked statistically significant differences compared to control conditions. Specifically, ING ranked relatively higher in maintaining core temperature (SUCRA = 67.0%), whereas topical application (Top) exhibited the weakest potential for thermoregulation (SUCRA = 6.2%) and might even lead to a slight upward trend in temperature due to the physical obstruction of heat dissipation.

**Conclusion:**

The interventional efficacy of menthol in the heat appears to be co-regulated by the administration route and the specific exercise task. While the current evidence does not establish definitive superiority among the administration routes due to the lack of statistical significance, ingestion may serve as an exploratory option for long-duration endurance events, whereas mouth rinsing could be considered for tasks focusing on instantaneous power output. Given that menthol may mask actual subjective thermal perception without alleviating objective physiological heat strain, its application must be combined with objective physiological monitoring in practice to ensure exercise safety.

**Systematic Review Registration:**

www.crd.york.ac.uk/prospero, identifier: CRD420261340546.

## 1 Introduction

Endurance exercise in hot or humid environments induces acute peripheral vasodilation and exacerbates cardiovascular strain, accompanied by a progressive rise in core body temperature. This thermal stress is widely believed to trigger the brain's “anticipatory regulation” mechanism, which preemptively reduces the recruitment of motor units by increasing the perception of fatigue, often leading to a significant impairment in exercise performance (1, 2). To mitigate heat stress, traditional physical cooling strategies—such as cold-water immersion, ice slurry ingestion (ING), or intermittent neck cooling—have been widely implemented (3–5). However, in field-based scenarios such as outdoor competitions or firefighting, these strategies are often limited by bulky equipment, logistical complexity, and potential gastrointestinal discomfort (6–9). Consequently, exploring more portable and efficient alternative strategies has become an urgent necessity in the field of exercise science.

In recent years, menthol has gained extensive attention as a promising ergogenic aid for enhancing exercise performance (10). From a neurophysiological perspective, menthol acts as an agonist for the cold-sensing receptor TRPM8, transmitting non-thermal “coolness” signals to the central nervous system. This is thought to “deceive” the brain's thermoregulatory center, thereby alleviating the subjective perception of thermal stress (11–13). Currently, menthol administration protocols exhibit distinct route-specificity, primarily involving three modes: ingestion, mouth rinsing (MR), and topical application (Top) (10).

Despite the reported ergogenic potential of menthol, a notable “disconnect” exists in the current literature: improvements in exercise performance are not consistently accompanied by reductions in objective physiological markers, such as core temperature or heart rate (14, 15). This decoupling suggests that menthol's efficacy may be fundamentally rooted in neurobiological modulation rather than classical thermodynamics. Theoretically, this phenomenon can be interpreted through the Psychobiological Model or the Central Governor Model, which posit that exercise intensity is regulated by the brain based on the interplay between afferent sensory feedback and conscious effort (16–18). By activating TRPM8-mediated cold receptors, menthol potentially offers a non-thermal “cool” signal that may dampen the perceived magnitude of thermal strain and the Rating of Perceived Exertion (RPE) (19–21). Consequently, this perceptual manipulation likely shifts the individual's pacing strategy, potentially allowing athletes to sustain a higher work rate despite maintaining, or even accumulating, a greater degree of objective physiological heat strain (21–23).

However, how this perception-based regulatory pathway varies across different administration routes—each targeting distinct sensory sites (e.g., oral mucosa vs. cutaneous receptors)—remains incompletely understood. Current evidence is largely derived from fragmented, route-specific studies, which typically employ pairwise comparisons against a placebo, making it difficult to systematically compare delivery strategies across diverse exercise tasks (22–24). Such a restricted focus may fail to fully account for the relative potency between active interventions, leaving a critical gap in determining the optimal delivery strategy for specific athletic demands. Crucially, the absence of “head-to-head” trials directly comparing routes like mouth rinsing and topical application limits the practical utility of conventional meta-analyses. To address this, Network Meta-Analysis (NMA) provides a robust framework to integrate both direct and indirect evidence within a unified analytical model. By leveraging a common comparator, NMA enables the generation of exploratory probabilistic rankings (e.g., SUCRA), offering a data-driven hierarchy of various menthol administration routes that is currently missing in the literature (25, 26).

Based on the hypothesis that different administration routes may exert “task-specific” effects, this study integrates Time Trial, Time to Exhaustion, and Mean Power Output into a unified analytical framework. By encompassing multiple dimensions—including work rate, exercise tolerance, and power production—this evaluation system aims to comprehensively reveal the potential efficacy of menthol across diverse exercise scenarios, thereby providing targeted supplementation recommendations for coaches and athletes.

## 2 Materials and methods

This study protocol has been registered at PROSPERO (Registration Number: CRD420261340546), specifying the research objectives, inclusion and exclusion criteria, interventions, control measures, and planned outcome assessments. The implementation of this systematic review strictly followed the pre-registered protocol and adhered to the Preferred Reporting Items for Systematic Reviews and Meta-Analyses (PRISMA) checklist for implementation and reporting (27).

### 2.1 Search strategy

We systematically searched PubMed, Embase, CINAHL, Web of Science, Scopus, Cochrane Library, CNKI, Wanfang, and VIP databases from inception to October 15, 2025. The search strategy combined MeSH terms and free text words, developed based on PubMed and adapted for other databases. Additionally, we manually searched the reference lists of all included studies to ensure maximum retrieval of relevant research. Details are shown in Table 1.

**Table 1:** Database search strategy.

Search combination	Search term	Search field
#1	Menthol OR “Peppermint Oil”	MeSH terms
#2	menthol OR “L-menthol” OR “peppermint oil” OR “menthol solution”	Title/Abstract
#3	endurance OR performance OR “time trial” OR “time to exhaustion” OR “power output” OR “exercise capacity”	MeSH terms
#4	“heart rate” OR “core temperature” OR “rectal temperature” OR “skin temperature” OR “thermoregulation” OR “physiological strain”	Title/Abstract
#5	heat OR hot OR “high temperature” OR “thermal stress” OR hyperthermia OR “environmental heat”	Publication type
#6	“Randomized Controlled Trial” OR “Randomized Controlled Trial” OR “RCT”	Title/Abstract
#7	(#1 OR #2) AND (#3 OR #4) AND (#5 OR #6)	Combined

### 2.2 Inclusion and exclusion criteria

Two reviewers independently screened the titles and abstracts of the literature, followed by a full-text review of potentially eligible articles to determine final inclusion. Any disagreements were resolved through discussion or third-party arbitration. Inclusion criteria were established based on the PICO-S framework: (1) Population (P): Healthy adult subjects (including athletes or fitness enthusiasts with regular exercise habits) regardless of gender; (2) Intervention (I): Any form of menthol administration, including but not limited to ingestion, mouth rinsing, or topical application; (3) Comparator (C): Blank control (e.g., room-temperature water and no intervention) or placebo control (e.g., placebo capsules and non-menthol topical agents); studies involving combined use of other supplements (e.g., caffeine and sodium bicarbonate) where protocols differed between groups were excluded; (4) Outcome (O): Reporting at least one of the following indicators: endurance performance (time trial, time to exhaustion, or average power) and physiological responses (heart rate or core temperature); (5) Study Design (S): Randomized Controlled Trials (RCT), including crossover or parallel-group designs; (6) Environmental conditions: Experiments must be conducted in heat (ambient temperature T≥25 °C). Exclusion criteria included: non-randomized controlled trials (e.g., reviews, case reports and animal studies), clinical diseases affecting performance or thermoregulation, incomplete data descriptions, mismatched interventions, and duplicate publications.

### 2.3 Data collection

Two researchers independently conducted literature screening and data extraction. The process involved an initial screening of titles and abstracts, followed by a full-text review to determine final inclusion, with any discrepancies resolved through discussion or by consulting a third party. Data were extracted using a pre-designed form, covering: (1) basic information: **first** author and year of publication; (2) participant characteristics: sample size, participant type (athletes or fitness enthusiasts), age, and gender; (3) intervention and environmental characteristics: environmental conditions (temperature in °C and relative humidity in %), administration protocols (route: ingestion [ING], mouth rinse [MR], or topical application [Top]; concentration in %; dosage; and frequency), and exercise tasks (mode: e.g., running or cycling; intensity; and protocol: time trial [TT], time-to-exhaustion [TTE], or constant power exercise); (4) outcome measures: exercise performance (mean and standard deviation [SD] for average power, TT completion time, or TTE) and physiological responses (mean and SD for heart rate [HR] and core temperature during exercise). For studies with incomplete data descriptions, the original authors were contacted; if unavailable, data were converted or estimated from standard errors (SE), confidence intervals (CI), or t-values according to Cochrane Handbook recommendations. For crossover design studies, data from the end of each intervention phase were extracted.

### 2.4 Risk of bias and certainty of evidence

The risk of bias in the included studies was evaluated using the Cochrane Risk of Bias tool for randomized trials (RoB 2) (28). The assessment encompassed five specific domains: (D1) bias arising from the randomization process, (D2) bias due to deviations from intended interventions, (D3) bias due to missing outcome data, (D4) bias in measurement of the outcome, and (D5) bias in selection of the reported result. Each domain was judged as “low risk of bias,” “some concerns,” or “high risk of bias,” followed by an overall risk of bias judgment for each study. The certainty of the evidence was further evaluated using the GRADE (Grading of Recommendations Assessment, Development and Evaluation) system (29, 30). The evidence was downgraded based on five criteria: risk of bias, inconsistency, indirectness, imprecision, and publication bias, and categorized into four levels: high, moderate, low, or very low. Two reviewers independently performed the quality assessment; any discrepancies were resolved through discussion or, if necessary, by consulting a third expert.

### 2.5 Data analysis

This study utilizes a network meta-analysis (NMA). Data synthesis was performed using the network Meta package in STATA 18.0 software. As the outcome measures are continuous variables, the effect size is expressed as the standardized mean difference (SMD) with its corresponding 95% confidence interval (CI), with the significance level set at α = 0.05. For crossover trials, to account for within-subject correlation, a conservative correlation coefficient of r = 0.5 was imputed to adjust the standard errors in accordance with the Cochrane Handbook guidelines, unless exact paired statistics were available. First, a network plot was constructed to illustrate the direct comparison relationships between various interventions. For closed-loop network structures, node-analysis techniques were employed for global inconsistency testing; if *P* > 0.05, consistency was considered satisfactory, and a consistency model was used for calculations. Simultaneously, local inconsistency was evaluated using the node-splitting method; a *P* < 0.05 indicated significant inconsistency. The relative efficacy of all interventions was ranked using the surface under the cumulative ranking curve (SUCRA). SUCRA values range from 0% to 100%, where a value closer to 100% indicates a higher probability that the intervention is the optimal measure. Potential publication bias was assessed using adjusted comparison-adjusted funnel plots and Egger's regression test.

To maximize statistical power and overcome the issue of limited sample sizes in individual study designs, different endurance metrics—specifically time trial (TT) completion time and time to exhaustion (TTE)—were pooled into a unified ‘exercise endurance' outcome domain. Although these testing protocols utilize different measurement scales, they are widely recognized as valid indicators of overall endurance capacity (31–33). Due to the opposite directionalities of these metrics, their data directions were uniformly aligned prior to data synthesis. The statistical appropriateness of combining these distinct continuous outcomes was further ensured by employing the standardized mean difference (SMD), which standardizes the data into a uniform, unitless scale to achieve effective quantitative synthesis. Notably, mean power output was evaluated as a distinct performance construct and analyzed in a separate network model.

## 3 Results

### 3.1 Literature identification and selection process

**Figure 1:**
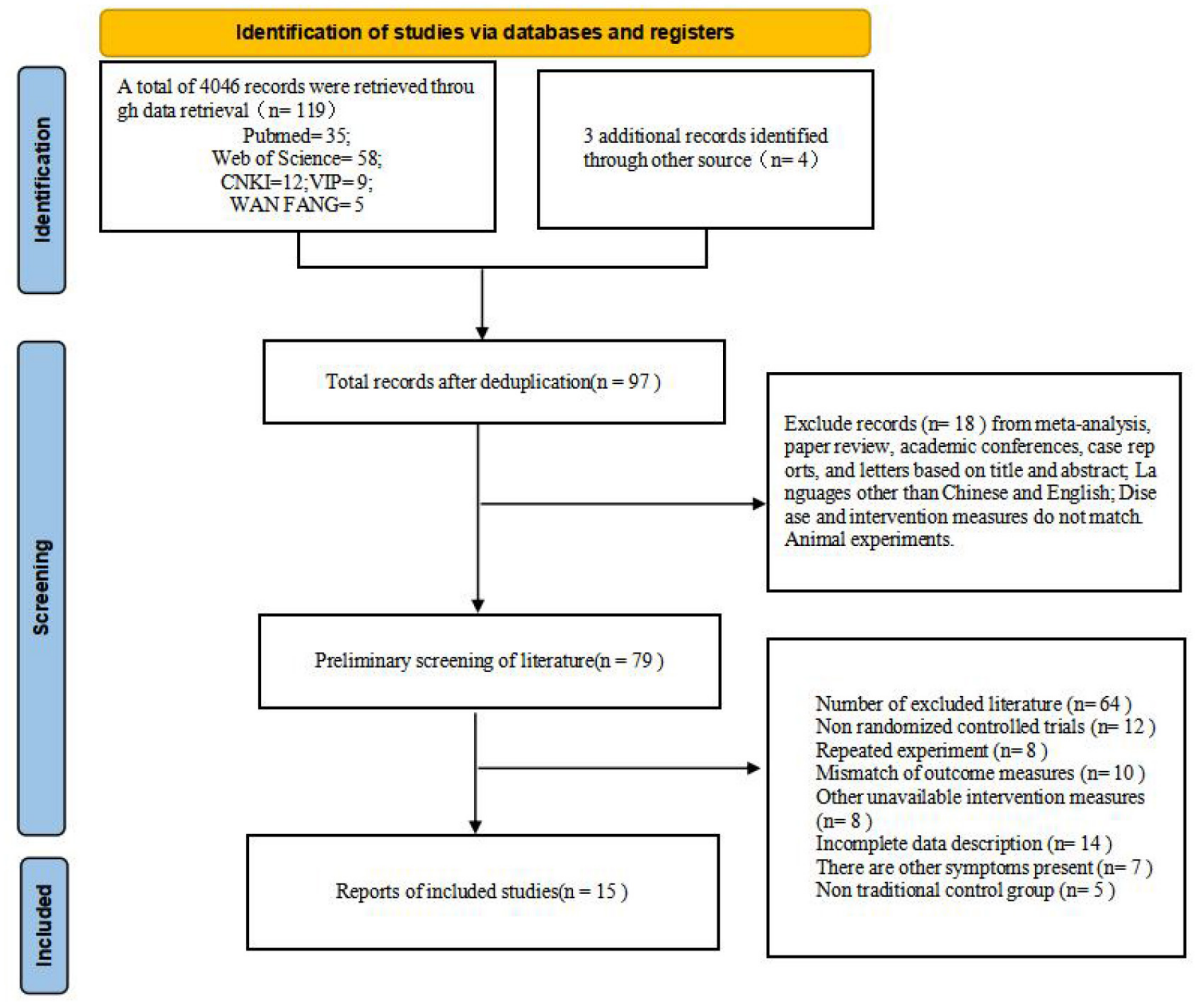
Preferred reporting items for systematic reviews and meta-analyses (PRISMA) flow diagram.

The literature search was conducted strictly in accordance with the protocol outlined in the PRISMA flow diagram (Figure 1). A total of 123 records were initially identified, consisting of 119 records retrieved from multiple databases—including PubMed (*n* = 35), Web of Science (*n* = 58), CNKI (*n* = 12), VIP (*n* = 9), and WANFANG (*n* = 5)—along with 4 additional records identified through other sources. After removing duplicates, 97 records remained for the screening process.

During the initial screening of titles and abstracts, 18 records were excluded for failing to meet inclusion criteria (e.g., reviews, conference papers, case reports, letters, non-Chinese/English languages, animal experiments, or mismatched diseases/interventions). Subsequently, a full-text assessment was performed on the remaining 79 studies, resulting in the exclusion of 64 records for the following specific reasons: non-randomized controlled trials (*n* = 12), repeated experiments (*n* = 8), mismatched outcome measures (*n* = 10), unavailable intervention measures (*n* = 8), incomplete data description (*n* = 14), presence of other symptoms (*n* = 7), and non-traditional control groups (*n* = 5). Ultimately, 15 studies met all inclusion criteria and were included in the systematic review and network meta-analysis. The detailed visualization of the selection process and results is shown in Figure 1.

### 3.2 Characteristics of included studies

The 15 included randomized controlled trials (RCTs) were published between 2010 and 2025 (6, 7, 34–46) (see Table 2), with a substantial proportion representing recent research from the last few years (34, 36, 43). The total sample size across all studies is approximately 165, with individual study samples ranging from 8 to 22, overall reflecting the small-sample characteristics typical of exercise physiology experiments. Participants were primarily healthy individuals with a training background, including trained cyclists, triathletes, runners, and physically active youth. To enhance the interpretability of our findings, specific fitness markers such as maximal oxygen uptake (VO2max) or peak oxygen uptake (VO2peak) and training status were explicitly extracted; the reported values ranged from 21.3 to 63.0 ml·kg^−1^·min^−1^, reflecting a generally high level of aerobic fitness among the cohorts. The gender distribution was predominantly male, although several studies (35, 38, 43) included female participants. The age of the subjects mainly ranged from 20 to 42 years, with only one study specifically targeting adolescent athletes aged approximately 16 (36).

**Table 2:** Characteristics of the included studies (Intervention details are categorized by route in Table 3).

Reference	Participants (*n*, Sex, Age)	Training status & Fitness level	Exercise protocol	Environmental conditions	Administration route	Outcomes
Lee et al. (7)	•8 Males •Age: 20.6 ± 1.3	•Non-professional athletes •End-exercise VO2 (with firefighter gear): 21.3–24.0 ml·kg^−1^·min^−1^	•Mode: Treadmill walking •Protocol: Initial 4 km/h, increased to 6 km/h and maintained for 30 min	Temp: 25.0 ± 0.5 °C, RH: 40 ± 3%	Topical spray	Core temperature, Heart rate
Barwood et al. (34)	•8 (7 M, 1 F) •Age: 36 ± 5	•Trained triathletes •>2 years of competitive experience (some at UK age-group national level)	•Mode: Swim-to-cycle •Protocol: 30-min swim (85% 400m PB) + 20-km cycling TT	Swim: 28.5 pm ± 0.3 °C (tropical water); Cycle: air temp ~28 °C, ~65% RH (WBGT “Red Flag” heat risk)	Topical gel	TT, Core temperature, Heart rate, Mean power output
Gavel et al. (35)	•9 Females •Age: 26.7± 1.4	•Trained •Trained ≥5 days/week in summer and participated in winter training •VO2max: 50.8 ± 6.0 ml·kg^−1^·min^−1^	•Mode: Indoor cycling •Protocol: 30-km individual TT (Self-paced, ~64% HRmax)	Temp: 30.2 °C; RH: 70%	Mouth rinse	TT, Heart rate, Mean power output
Hawke et al. (36)	•11 Males •Age: 16 ± 1.3	•Trained •Recruited from local cycling clubs •VO2peak: 62.97 ± 7.47 ml·kg^−1^·min^−1^	•Mode: Cycling •Protocol: Modified 30-min variable-intensity test (Five 6-min cycles with sprints)	Temp: 31.4 ± 0.9 °C; RH: 23.4% ± 3.7%	Mouth rinsing	TT, Mean power output
Stevens et al. (10)	•11 Males •Age: 30± 9	•Trained •Average weekly running distance: 44 ± 20 km •VO2peak: 60.2 ± 6.8 ml·kg^−1^·min^−1^	•Mode: Non-motorized treadmill (NMT) running •Protocol: 20-min pre-load (70% VO2max) + 3-km maximal TT	Temp: 32.5 ± 0.1 °C; RH: 46.8 ± 7.9%	Mouth rinsing	TT
Galpin et al. (37)	•13 Males •Age: 25.3 ± 5.0	•Trained •Average 5.3 ± 1.8 h/week of high-intensity exercise •VO2max: 54.8 ± 6.2 ml·kg^−1^·min^−1^	•Mode: Cycle ergometer •Protocol: 2 × 5-min high-intensity bouts (50% PPO) + TTE test (30% PPO)	Temp: 25.0 ± 1.0 °C; RH: 53.0% ± 1.0%	Topical spray	TTE
Parton et al. (38)	•22 (11 M, 11 F) •Age: 20 ± 1 (M), •22 ± 2 (F)	•Trained •VO2peak: 63.0 ± 7.5 ml·kg^−1^·min^−1^ (Males); 50.8 ± 6.0 ml·kg^−1^·min^−1^ (Females)	•Mode: Cycle ergometer •Protocol: Fixed-RPE protocol (RPE set at 16 on Borg 6–20 scale)	Temp: 34.9 ± 0.5 °C; RH: 40.6 ± 2.2%	Mouth rinse	TTE
Flood et al. (39)	•8 Males •Age: 26± 5	•Trained •Habituated to endurance exercise •VO2peak: 52.7 ± 6.3 ml·kg^−1^·min^−1^	•Mode: Cycle ergometer •Protocol: Fixed-RPE protocol (RPE set at 16 on Borg 6–20 scale)	Temp: 35.0 ± 0.8 °C; RH: 47.8 ± 2.3%	Mouthwash	TTE, Mean power output
Hermand et al. (40)	•13 Males •Age: 21 ± 4	•Trained •Regularly participating in endurance training •VO2peak: 51.0 ± 8.2 ml·kg^−1^·min^−1^	•Mode: Outdoor running •Protocol: 10-km all-out TT	Temp: 29.0 ± 1.3 °C; RH: 59.0 ± 13.6%	Topical	TT
Riera et al. (41)	•12 Males •Age: 42 ±13	•Trained •Average weekly cycling distance: 250 ± 82 km •VO2max: 57.0 ± 10.3 ml·kg^−1^·min^−1^	•Mode: Cycling •Protocol: 20-km all-out TT (Resistance set at second ventilatory threshold, ~335 W)	Temp: 30.7 ± 0.8 °C; RH: 78 ± 0.03%	Ingestion	TT
Tran Trong et al. (42)	•10 Males •Age: 25 ± 5	•Trained •Average weekly cycling distance: 250 ± 82 km •VO2max: 57.0 ± 10.3 ml·kg^−1^·min^−1^	•Mode: Outdoor running •Protocol: 5-km all-out TT	Temp:33.1 ± 1.1 °C; RH: 62.0 ± 4.0%	Ingestion	TT
Bray et al. (43)	•10 (5 M, 5 F) •Age: 23 ± 5	•Trained •Recreationally trained cyclists or triathletes •VO2max: 52.3 ± 8.6 ml·kg^−1^·min^−1^	•Mode: Indoor cycling •Protocol: 40-min pre-load (50% VO2max) + 15-min self-paced TT	Temp: ~35.0 °C, RH: ~54%	Ingestion	Core temperature, Heart rate, Mean power output
Stevens et al. (44)	•11 Males •Age: 29± 9	•Trained •Average weekly running distance: 44 ± 20 km •VO2peak: 60.2 ± 6.8 ml·kg^−1^·min^−1^	•Mode: NMT running •Protocol: 5-km self-paced all-out TT	Temp: 33 °C; RH: 46 ± 5.7%	Mouth rinse	TT, Core temperature
Mündel and Jones (45)	•9 Males •Age: 25 ± 7	•Trained •Healthy individuals regularly performing physical exercise •VO2max: 54.5 ± 5.0 ml·kg^−1^·min^−1^	•Mode: Cycling •Protocol: TTE test at fixed intensity (65% MAP)	Temp: 34 ± 1 °C; RH: 27 ± 4%	Mouth rinse	TTE
Jeffries et al. (46)	•10 Males •Age: 33 ± 9	•Trained •Frequently participating in endurance training •VO2peak: 53.0 ± 8.2 ml·kg^−1^·min^−1^	•Mode: Cycle ergometer •Protocol: TTE test at fixed intensity (70% Wmax)	Temp: 35 ± 0.2 °C; RH: 40 ± 0.5%	Mouth rinse	TTE

Regarding experimental design, all exercise interventions and tests were conducted under high-temperature or specific heat-stress conditions. Ambient temperatures were set between 25.0 °C and 35.0 °C (with the majority exceeding 30.0 °C), and relative humidity ranged from 23.4% to 78.0% to simulate various levels of heat-humidity stress. The exercise protocols encompassed non-motorized or conventional treadmill running, outdoor running, indoor stationary cycling, and multi-sport events combining swimming and cycling (34). Performance assessments were categorized into three primary types: time trials (TT) based on fixed distance or work (e.g., 3–20 km all-out performance (6, 41)), constant-load time-to-exhaustion tests (TTE (45)), and fixed Rating of Perceived Exertion (RPE) protocols (39). All included studies measured relevant endurance performance metrics (TT, TTE, or mean power output), and the majority simultaneously monitored core body temperature and heart rate to evaluate the comprehensive impact of menthol on thermophysiological responses.

The menthol administration protocols across the experimental groups exhibited significant route-specificity and dose diversity. While Table 2 provides a general overview of the interventions, the specific concentrations, dosages, and application timings for each administration route—primarily encompassing mouth rinsing, ingestion, and topical application—are comprehensively detailed in Table 3 to facilitate a clearer comparison of the dosing strategies. MR was the most frequently utilized intervention route (6, 35, 36, 38, 39, 44–46), with the majority of studies employing a 0.01% L-menthol solution. This protocol typically involved periodic swilling for 5 to 10 s before and during exercise followed by expectoration, with swallowing strictly prohibited. Ingestion was adopted in three studies, with concentrations ranging from 0.01% to 0.5% (41–43); participants generally consumed the solution in phases, including a hybrid “swill-then-swallow” format (43). Topical application was employed in four studies and featured the most diverse delivery media. These included 0.8% or 8% menthol sprays applied to the whole body or the posterior neck (7, 37), 3.5% menthol gel (34), and T-shirts soaked in 4% menthol solution (40). These interventions were typically implemented via localized or extensive physical contact during pre-exercise or recovery intervals.

**Table 3:** Detailed characteristics of menthol doses and administration protocols.

Reference	Route	Concentration (%)	Formulation	Application site/Method	Total dose/Frequency
Ingestion (ING)	
Riera et al. (41)	Ingestion	0.5%	Menthol solution	Gastrointestinal tract	190 mL per serving (1,140 mL total); every 5 km
Tran Trong et al. (42)	Ingestion	0.05%	Menthol solution	Gastrointestinal tract	190 mL per serving (570 mL total); 3 doses
15.6-7.4,-14.3499ptBray et al. (43)	Ingestion	0.01%	Menthol beverage	Gastrointestinal tract	85 mL per dose (510 mL total); every 10 min
Mouth Rinsing (MR)	
Gavel et al. (35)	Mouth rinse	~0.1%	L-menthol solution	Oral cavity (5-s swill & expectorate)	25 mL; pre-exercise & every 5 km (7 times)
Hawke et al. (36)	Mouth rinse	0.01%	L-menthol solution	Oral cavity (5-s swill & expectorate)	25 mL; 60-s pre-start & each cycle (6 times)
Stevens et al. (6, 10)	Mouth rinse	0.01%	L-menthol solution	Oral cavity (5-s swill & expectorate)	25 mL; 60-s pre-start & each cycle (6 times)
Parton et al. (38)	Mouth rinse	0.01%	L-menthol solution	Oral cavity (30-s swill & expectorate)	25 mL; 30-s pre-exercise & every 10 min
Flood et al. (39)	Mouth rinse	0.01%	L-menthol solution	Oral cavity (5-s swill & expectorate)	25 mL; 1.5 min pre-exercise & every 10 min
Stevens et al. (44)	Mouth rinse	0.01%	L-menthol solution	Oral cavity (5-s swill & expectorate)	25 mL; every 1 km during trial
Mündel & Jones (45)	Mouth rinse	0.01%	L-menthol solution	Oral cavity (10-s swill & expectorate)	25 mL; every 10 min during exercise
15.6-7.4,-14.3499ptJeffries et al. (46)	Mouth rinse	0.01%	L-menthol solution	Oral cavity (swill & expectorate)	25 mL; administered at 85% of baseline TTE
Topical (Top)	
Galpin et al. (37)	Topical	8%	Menthol spray	Posterior neck (wiped after 1-min)	2–4 mL; start of each rest interval
Hermand et al. (40)	Topical	4%	Soaked T-shirt	Torso skin	250–300 g solution; pre-start & every 2 km
Barwood et al. (34)	Topical	3.5%	Menthol gel	Torso and limbs	40 g (40 mL); applied 3 min prior to exercise
Lee et al. (7)	Topical	0.8%	Menthol spray	Whole body (excluding face/neck)	126 ± 9 g; once (10-min rest post-spray)

### 3.3 Literature quality assessment

**Figure 2:**
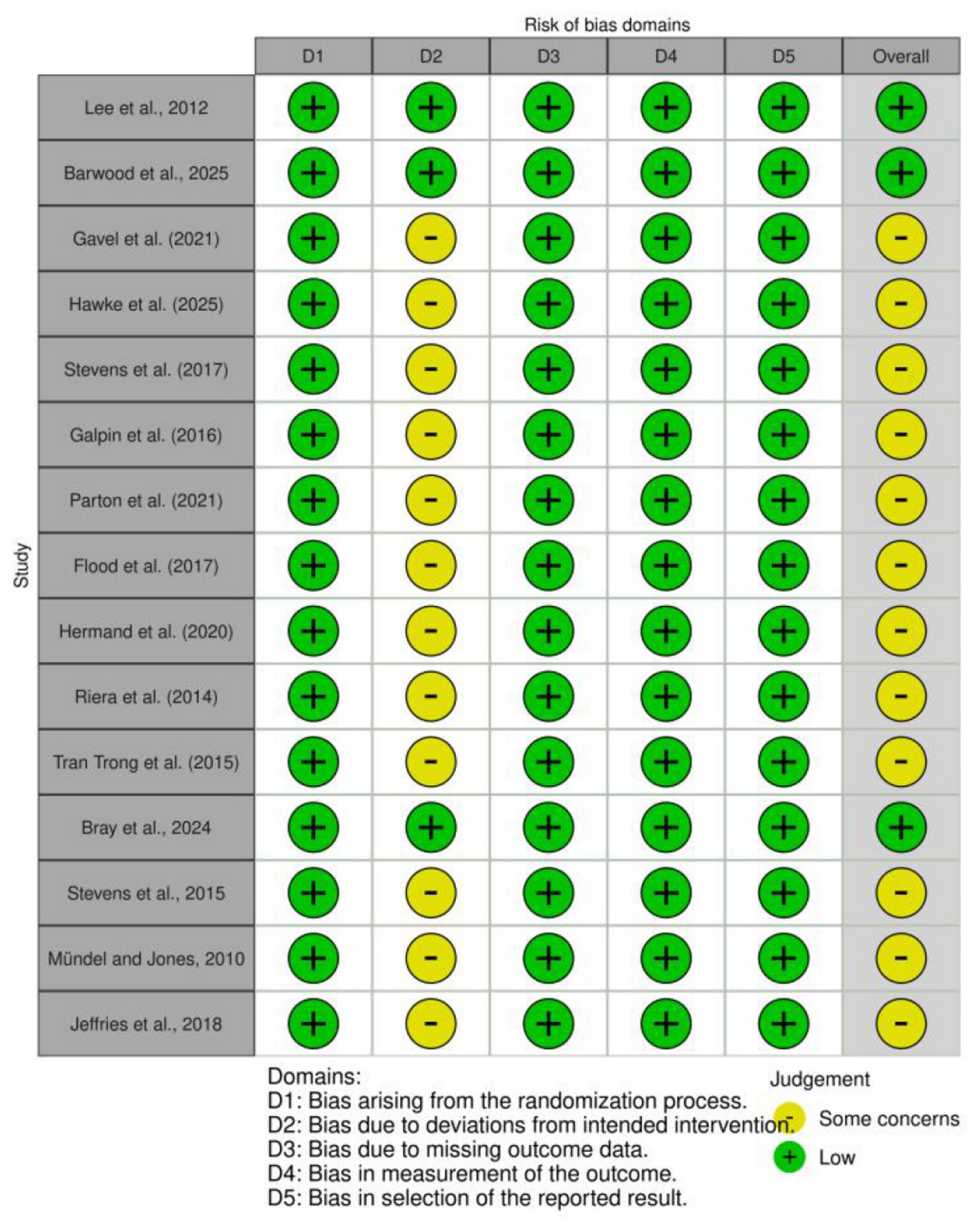
Risk of bias summary: review of the authors judgments about each risk of bias item for each included study.

**Figure 3:**
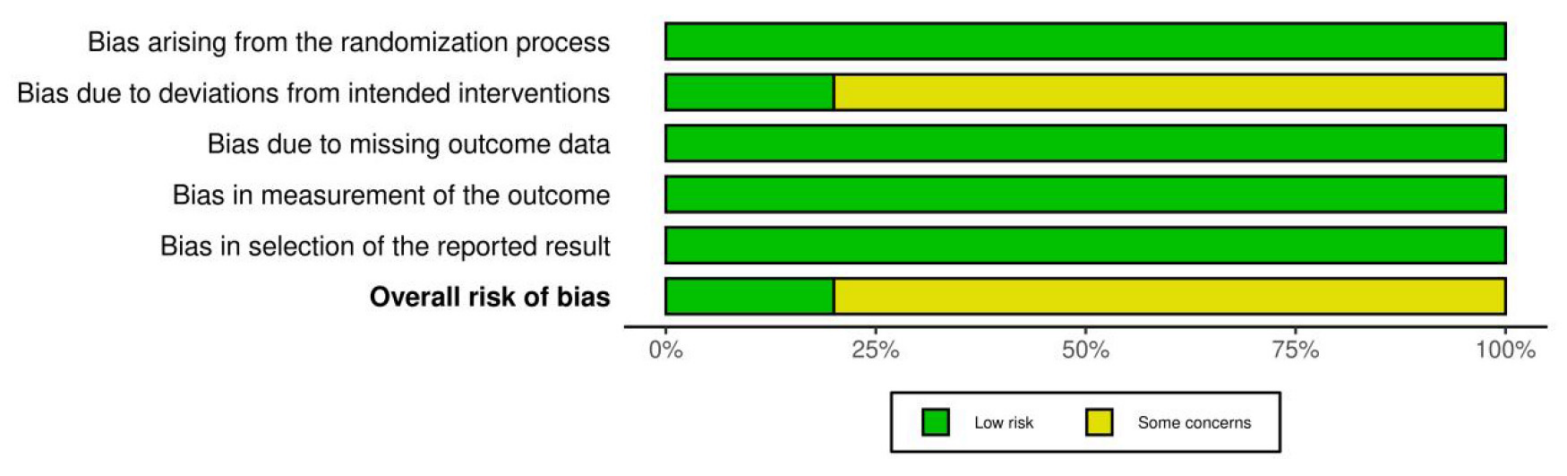
Risk of bias graph: review authors' judgments about each risk of bias item, presented as percentage of included studies.

We conducted a quality assessment of the 15 included randomized controlled trials using the Cochrane RoB 2.0 tool. Overall, the methodological quality of the studies was mostly judged as having “some concerns” or being at “low risk.” The risk of bias was primarily concentrated in the “deviations from intended interventions (D2)” domain. This is mainly attributed to the inherent difficulty in achieving complete double-blinding of both participants and researchers during the implementation of menthol interventions, such as mouth rinsing or topical application. In contrast, the risks associated with the randomization process (D1), missing outcome data (D3), and selective reporting (D5) were generally low (Figures 2, 3).

### 3.4 Analysis of exercise endurance outcomes

#### 3.4.1 Network plot of included studies

**Figure 4:**
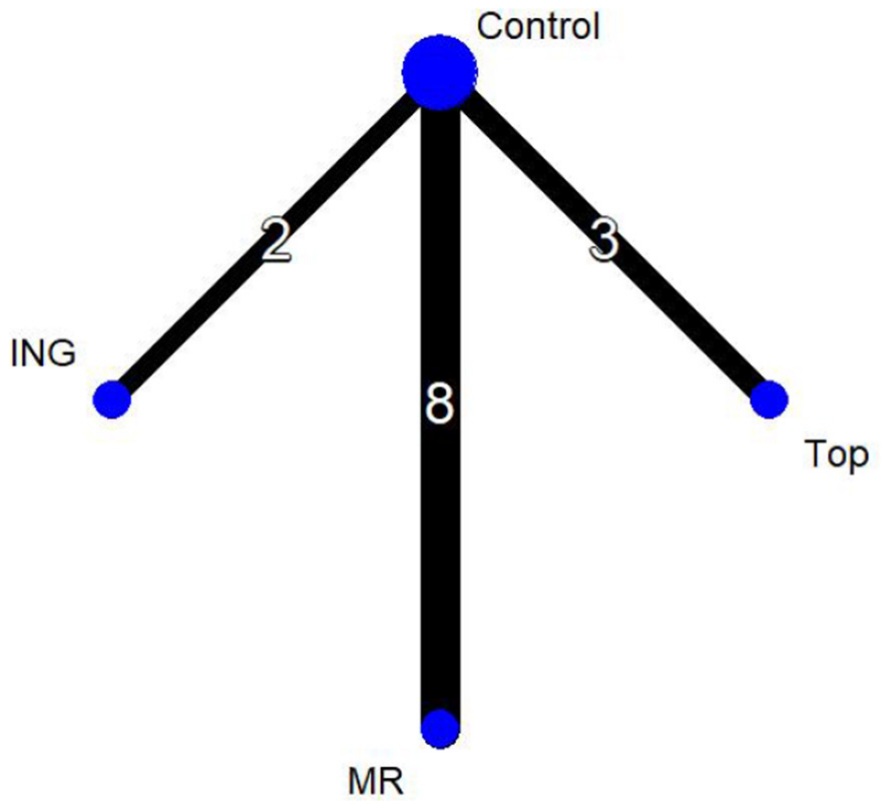
Network plot of endurance performance.

In Figure 4, the four nodes represent three distinct intervention measures. The connections between the nodes indicate direct comparisons. The administration routes include ingestion, mouth rinsing, and topical application, with the control group (Control) consisting of either a placebo or room-temperature water. In the network diagram, the thickness of the lines reflects the frequency of direct analytical comparisons between pairs of interventions. Since this network diagram contains no closed loops, a global consistency model was selected for the analysis.

Thirteen studies evaluated endurance performance and were included in the network meta-analysis (*n* = 272 participants, three intervention routes). The figure presents the evidence network for endurance performance comparisons, where the three intervention routes—all involving menthol—are compared against the control group (Control).

#### 3.4.2 Ranking of interventions

**Figure 5:**
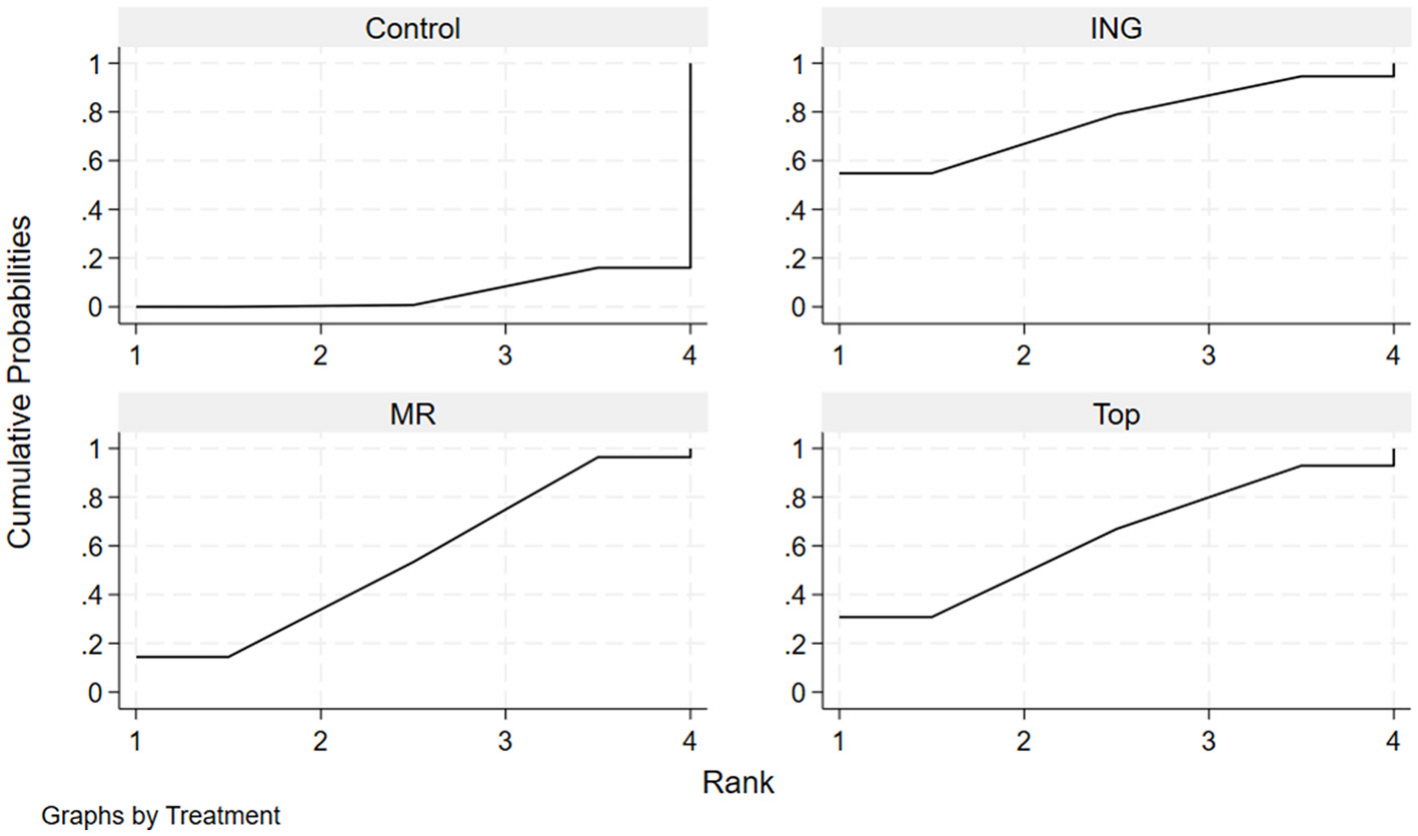
Cumulative ranking probability plots for endurance performance.

The effectiveness of each administration route in enhancing endurance performance in the heat was evaluated by calculating the surface under the cumulative ranking curve (SUCRA) and ranking probabilities (Table 4, Figure 5). The results indicated that ingestion had the highest probability (estimated SUCRA of approximately 76.1%) of being the optimal administration route for improving endurance performance in high-temperature environments. This was followed by topical application (SUCRA = 63.5%) and mouth rinsing (SUCRA = 54.7%). The cumulative ranking probability plots (Figure 5) visually demonstrate this ranking characteristic: Ingestion had a substantially higher probability of being ranked first compared to the other routes, while the Control curve lagged notably, with the majority of studies ranking it last (MeanRank = 3.8).

**Table 4:** SUCRA values for endurance performance outcomes.

Treatment	Sucra	Prbest	Meanrank
Control	5.6	0.0	3.8
ING	76.1	54.8	1.7
MR	54.7	14.4	2.4
Top	63.5	30.8	2.1

#### 3.4.3 League table analysis for exercise endurance

The league table (Table 5) presents the effect sizes (Standardized Mean Difference [SMD]) and their corresponding 95% Confidence Intervals (CIs) for the network meta-analysis across all administration routes. Pairwise comparisons from the network meta-analysis revealed that although all three active intervention routes exhibited a superior trend compared to the control group, the 95% CIs for all comparisons crossed the null point (0), indicating that these differences did not reach statistical significance: topical application showed the largest point estimate of effect size relative to the control group (SMD = 0.48, 95% CI [−0.12, 1.08]). The improvement effects for ingestion (SMD = 0.20, 95% CI [−0.48, 0.88]) and mouth rinsing (SMD = 0.12, 95% CI [−0.65, 0.89]) were similarly modest. In the mutual comparisons between different active intervention routes, the trend for Top being superior to MR (SMD = 0.36, 95% CI [−0.12, 0.84]) and ING (SMD = 0.28, 95% CI [−0.03, 0.59]) was most pronounced, though these also failed to reach statistical significance.

**Table 5:** League table of network meta-analysis for endurance performance.

Top	MR	ING	Control
Top	0.36 (−0.12, 0.84)	0.28 (−0.03, 0.59)	0.48 (−0.12, 1.08)
−0.36 (−0.84, 0.12)	MR	−0.08 (−0.66, 0.49)	0.12 (−0.65, 0.89)
−0.28 (−0.59, 0.03)	0.08 (−0.49, 0.66)	ING	0.20 (−0.48, 0.88)
−0.48 (−1.08, 0.12)	−0.12 (−0.89, 0.65)	−0.20 (−0.88, 0.48)	Control

### 3.5 Analysis of mean power outcomes

#### 3.5.1 Network plot of included studies

**Figure 6:**
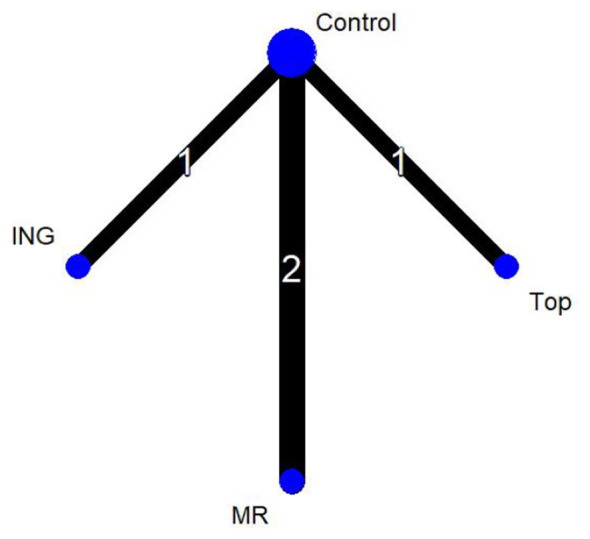
Network plot of mean power output.

In Figure 6, the four nodes represent three distinct intervention measures. The connections between the nodes indicate direct comparisons. The administration routes include ingestion, mouth rinsing, and topical application, with the control group (Control) consisting of either a placebo or room-temperature water. In the network diagram, the thickness of the lines reflects the frequency of direct analytical comparisons between pairs of interventions. Since this network diagram contains no closed loops, a global consistency model was selected for the analysis.

Four studies evaluated mean power output and were included in the network meta-analysis (*n* = 72 participants, three intervention routes). The figure presents the evidence network for these comparisons, where the three intervention routes—all involving menthol—are compared against the control group (Control).

#### 3.5.2 Ranking of interventions

**Figure 7:**
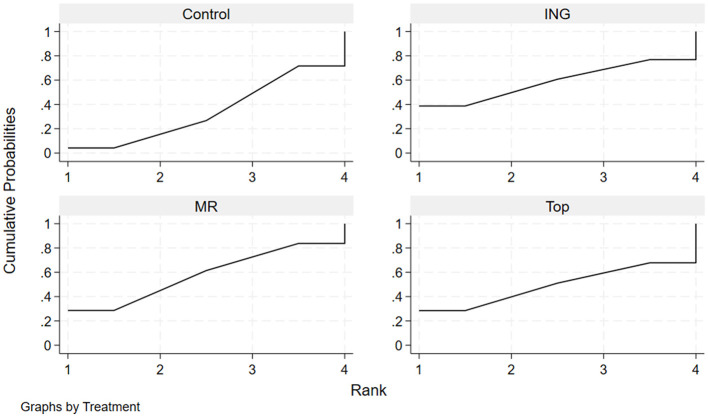
Cumulative ranking probability plots for mean power output.

The effectiveness of each administration route in enhancing mean power output in the heat was evaluated by calculating the surface under the cumulative ranking curve (SUCRA) and ranking probabilities (Table 6, Figure 7). The results indicated that for improving mean power output, mouth rinsing demonstrated the greatest potential for improvement, with the highest SUCRA value of approximately 66.9%. This was followed by topical application with a SUCRA value of 53.9%. In contrast, the performance of ingestion (SUCRA = 39.6%) for this indicator was equal to that of the control group (SUCRA = 39.6%). The cumulative ranking probability plots (Figure 7) further confirmed this trend: the MR curve showed the most rapid increase in cumulative probability at the first and second ranking positions, suggesting a high probability advantage as the optimal route for enhancing power output.

**Table 6:** SUCRA values and rankings for mean power output.

Treatment	Sucra	Prbest	Meanrank
Control	39.6	5.7	2.8
ING	39.6	20.4	2.8
MR	66.9	38.3	2.0
Top	53.9	35.6	2.4

#### 3.5.3 League table analysis for mean power

The league table (Table 7) presents the effect sizes (Standardized Mean Difference [SMD]) and their corresponding 95% CIs for the network meta-analysis across different administration routes. Pairwise comparisons from the network meta-analysis revealed that although MR and Top demonstrated advantages in the ranking, no active intervention route showed a statistically significant difference compared to the control group (*P* > 0.05): ingestion exhibited the largest point estimate of effect size relative to the control group (SMD = 0.23, 95% CI [−0.79, 1.26]), but the certainty of its improvement effect was low due to the extremely wide confidence interval. The effect size for mouth rinsing relative to the control group was 0.16 (95% CI [−1.15, 1.48]). Mutual comparisons between the active intervention routes (e.g., MR vs. Top) similarly showed that the 95% CIs crossed the null point (0), indicating that the differences in efficacy for power output between different routes were not statistically significant.

**Table 7:** League table of the network meta-analysis for mean power output.

Top	MR	ING	Control
Top	0.11 (−0.87, 1.09)	0.18 (−0.34, 0.71)	−0.05 (−0.93, 0.83)
−0.11 (−1.09, 0.87)	MR	0.07 (−1.04, 1.18)	−0.16 (−1.48, 1.15)
−0.18 (−0.71, 0.34)	−0.07 (−1.18, 1.04)	ING	−0.23 (−1.26, 0.79)
0.05 (−0.83, 0.93)	0.16 (−1.15, 1.48)	0.23 (−0.79, 1.26)	Control

### 3.6 Analysis of core temperature outcomes

#### 3.6.1 Network plot of included studies

**Figure 8:**
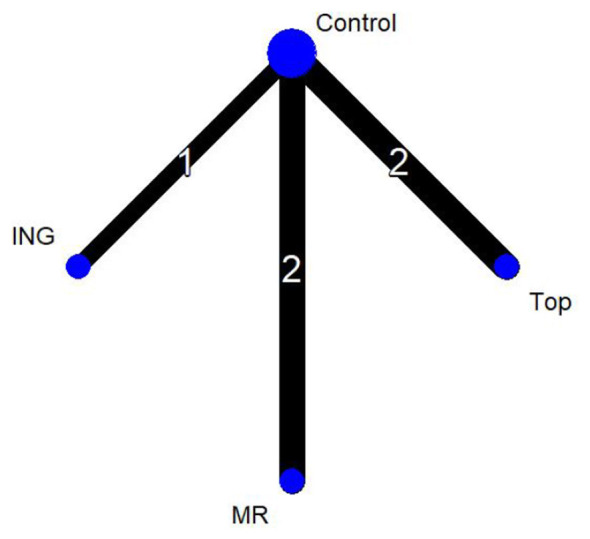
Network plot of core temperature.

In Figure 8, the four nodes represent three distinct intervention measures. The connections between the nodes indicate direct comparisons. The administration routes include ingestion, mouth rinsing, and topical application, with the control group (Control) consisting of either a placebo or room-temperature water. In the network diagram, the thickness of the lines reflects the frequency of direct analytical comparisons between pairs of interventions. Since this network diagram contains no closed loops, a global consistency model was selected for the analysis.

Five studies evaluated core temperature and were included in the network meta-analysis (n = 86 participants, three intervention routes). The figure presents the evidence network for these comparisons, where the three intervention routes—all involving menthol—are compared against the control group (Control).

#### 3.6.2 Ranking of interventions

**Figure 9:**
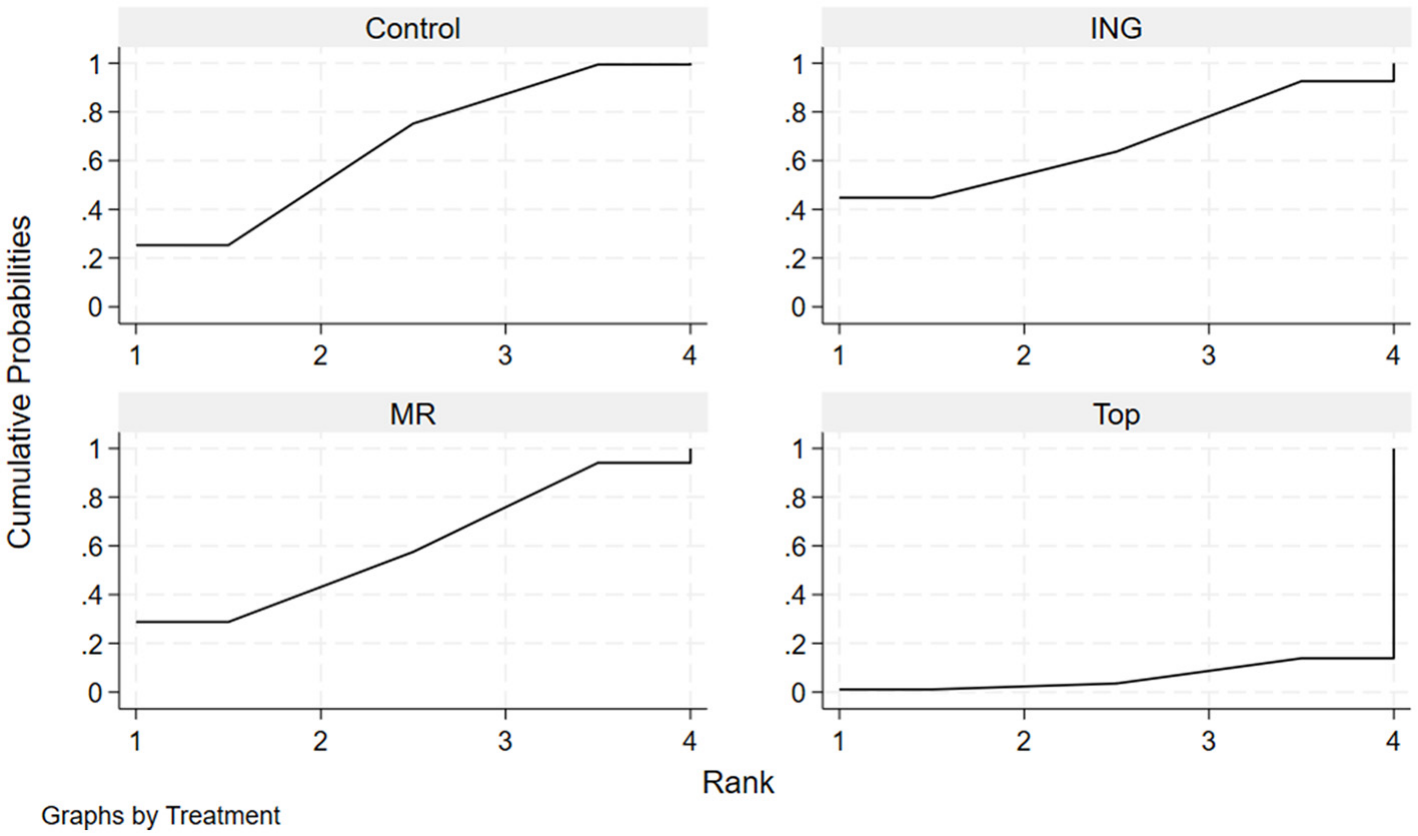
Cumulative ranking probability plots for core temperature.

The effectiveness of each administration route in maintaining or regulating core temperature in the heat was evaluated by calculating the surface under the cumulative ranking curve (SUCRA) and ranking probabilities. The results indicated that for maintaining or regulating core temperature, ingestion (SUCRA = 67.0%) and the control group (Control) (SUCRA = 66.6%) demonstrated a high degree of consistency, with both achieving a MeanRank of 2.0. This suggests that the physiological impact of menthol ingestion on core temperature may not provide a notable advantage over natural heat dissipation conditions. Mouth rinsing ranked third with a SUCRA value of 60.1%. In contrast, the SUCRA value for topical application was extremely low (only 6.2%), indicating that its potential for reducing or controlling the rise in core temperature was the weakest among all routes. The cumulative ranking probability plots (Table 8, Figure 9) further confirmed this distribution characteristic: the cumulative probability curves for ING, Control, and MR were relatively concentrated and rose rapidly across the first to third ranking positions. Meanwhile, the curve for Top only showed a sharp surge at the fourth ranking position, with a MeanRank of 3.8, representing a very high probability of being judged as the least effective administration route.

**Table 8:** SUCRA values for core temperature outcomes.

Treatment	Sucra	Prbest	Meanrank
Control	66.6	25.3	2.0
ING	67.0	44.8	2.0
MR	60.1	28.8	2.2
Top	6.2	1.1	3.8

#### 3.6.3 League table analysis for core temperature

The league table (Table 9) presents the effect sizes expressed as Standardized Mean Differences (SMD) and their corresponding 95% Confidence Intervals (CI) for the network meta-analysis of core temperature. Pairwise comparisons revealed that for core temperature regulation, most comparisons between administration routes, as well as between active interventions and the control group, failed to reach statistical significance. Specifically, none of the improvements relative to the control group were statistically significant, including ingestion (SMD = 0.13, 95% CI [−1.73, 1.99]), mouth rinsing (SMD = 1.41, 95% CI [−0.60, 3.41]), and topical application (SMD = −0.07, 95% CI [−1.59, 1.45]). In terms of inter-route comparisons, a significant difference was observed between Top and MR (SMD = 1.34, 95% CI [0.03, 2.64]), suggesting that MR possesses a superior trend in temperature control compared to Top. Furthermore, the negligible effect size of 0.13 between ING and the control further corroborates the equivalence of their efficacy in this outcome.

**Table 9:** League table of the network meta-analysis for core temperature.

Top	MR	ING	Control
Top	1.34 (0.03, 2.64)	0.06 (−1.01, 1.13)	−0.07 (−1.59, 1.45)
−1.34 (−2.64, −0.03)	MR	−1.28 (−2.97, 0.41)	−1.41 (−3.41, 0.60)
−0.06 (−1.13, 1.01)	1.28 (−0.41, 2.97)	LNG	−0.13 (−1.99, 1.73)
0.07 (−1.45, 1.59)	1.41 (−0.60, 3.41)	0.13 (−1.73, 1.99)	Control

### 3.7 Analysis of heart rate outcomes

#### 3.7.1 Network plot of included studies

**Figure 10:**
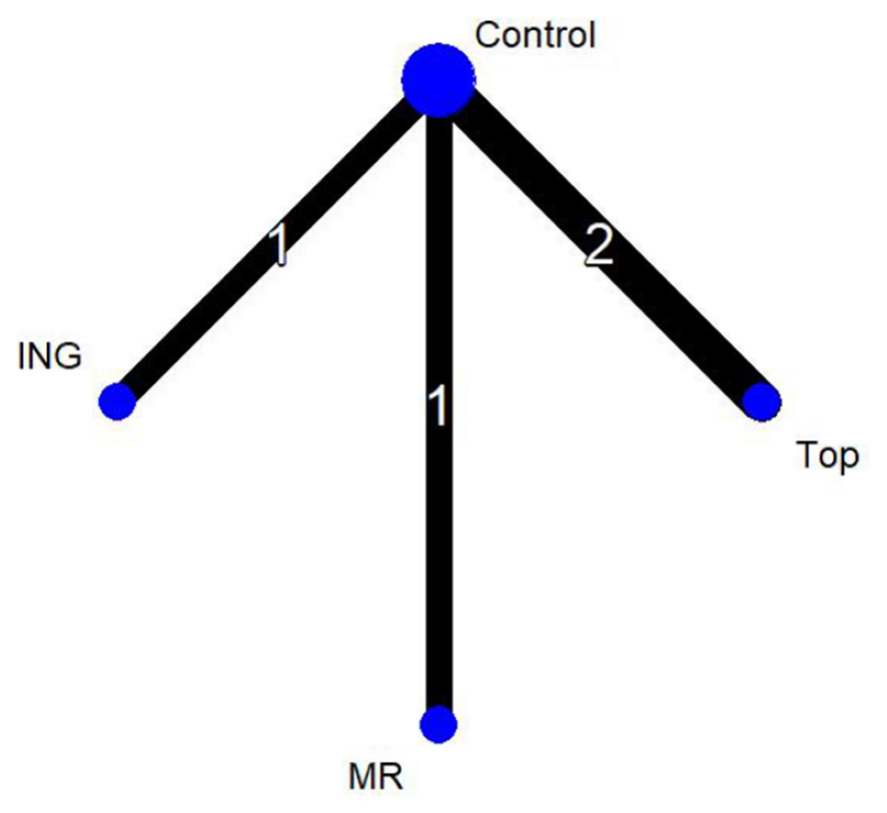
Network plot of heart rate.

In Figure 10, the four nodes represent three distinct intervention measures. The connections between the nodes indicate direct comparisons. The administration routes include ingestion, mouth rinsing, and topical application, with the control group (Control) consisting of either a placebo or room-temperature water. In the network diagram, the thickness of the lines reflects the frequency of direct analytical comparisons between pairs of interventions. Since this network diagram contains no closed loops, a global consistency model was selected for the analysis.

Four studies evaluated heart rate and were included in the network meta-analysis (*n* = 70 participants, three intervention routes). The figure presents the evidence network for heart rate comparisons, where the three intervention routes—all involving menthol interventions—are compared against the control group (Control).

#### 3.7.2 Ranking of interventions

**Figure 11:**
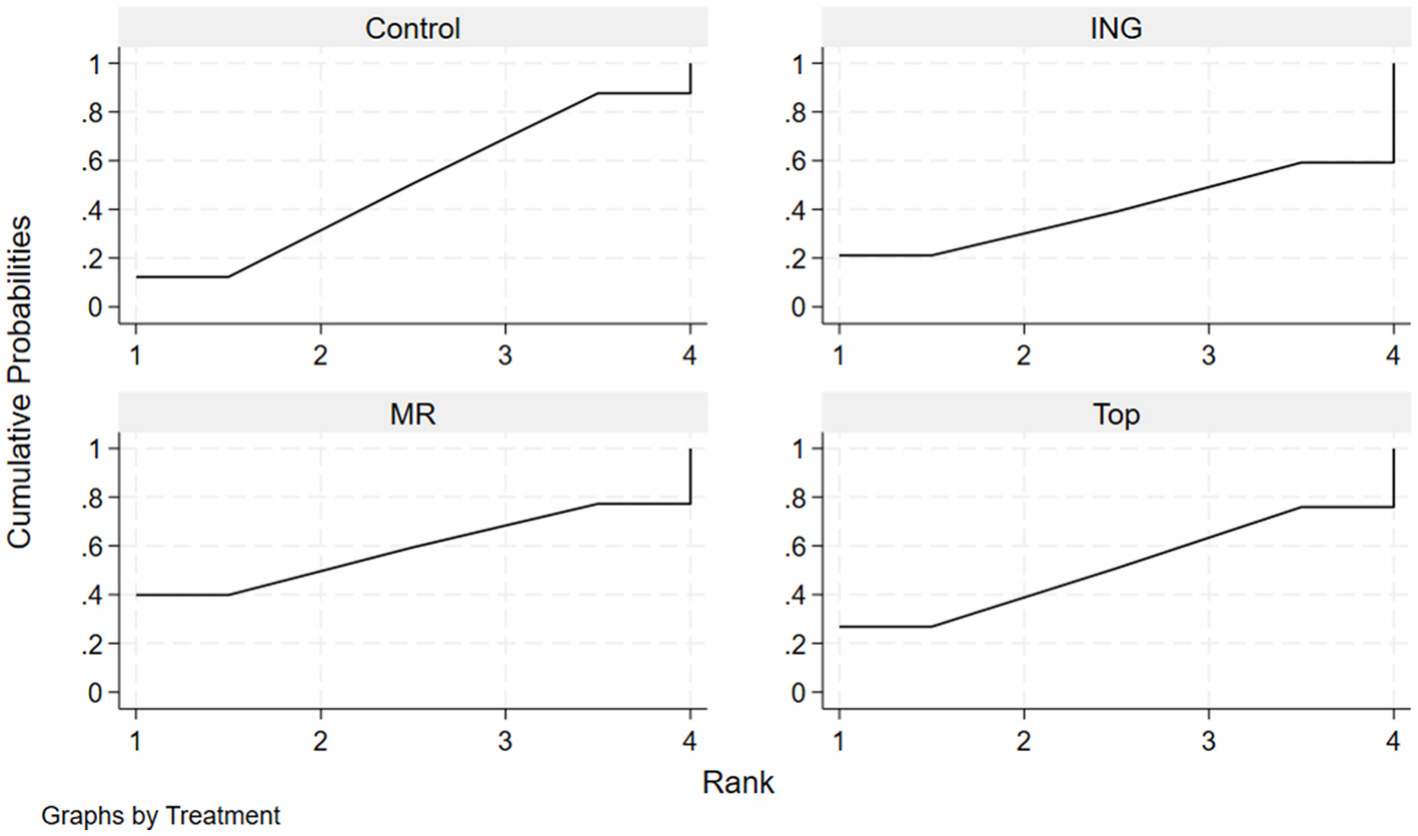
Cumulative ranking probability plots for heart rate.

The effectiveness of each administration route in regulating heart rate during exercise in the heat was evaluated by calculating the surface under the cumulative ranking curve (SUCRA) and ranking probabilities (Table 10, Figure 11). The results indicated that for heart rate regulation, mouth rinsing had the highest probability of being the optimal intervention (SUCRA = 58.8%), with a 39.8% probability of being ranked first (PrBest). Topical application (SUCRA = 51.2%) and the control group (Control) (SUCRA = 50.2%) demonstrated nearly identical performance, with both achieving a MeanRank of 2.5. Notably, ingestion yielded the lowest SUCRA value (39.8%) and a MeanRank of 2.8, suggesting that its performance in reducing heart rate during exercise in the heat may be inferior to other intervention methods or even the control. The cumulative ranking probability plots (Figure 11) visually reflected this trend: the MR curve had the highest starting point at the first ranking position and a faster upward slope, demonstrating a distinct ranking advantage. Conversely, the trajectories for Top and Control were nearly overlapping, further corroborating their equivalent efficacy. The ING curve showed higher cumulative probabilities at later ranking positions, indicating a relatively weaker regulatory effect.

**Table 10:** SUCRA values for heart rate outcomes.

Treatment	Sucra	Prbest	Meanrank
Control	50.2	12.2	2.5
ING	39.8	21.1	2.8
MR	58.8	39.8	2.2
Top	51.2	26.8	2.5

#### 3.7.3 League table analysis for heart rate

The league table (Table 11) presents the standardized mean differences (SMD) and corresponding 95% CIs for heart rate regulation effects across the various administration routes. Pairwise comparisons from the network meta-analysis revealed that menthol administration has a negligible impact on heart rate during exercise in the heat, with no route demonstrating a statistically significant difference compared to the control group (*P* > 0.05). Specifically, when comparing active interventions to the control, the regulatory effect sizes for both mouth rinsing (SMD = 0.12, 95% CI [−0.91, 1.16]) and ingestion (SMD = −0.13, 95% CI [−1.12, 0.87]) were extremely small, with confidence intervals widely crossing the null point (0). Furthermore, inter-route comparisons between the active pathways themselves also showed confidence intervals crossing the null point, indicating that differences in physiological heart rate feedback among different administration routes lack statistical significance.

**Table 11:** League table for heart rate in the network meta-analysis.

Top	MR	ING	Control
Top	−0.12 (−1.42, 1.18)	0.13 (−1.13, 1.40)	0.00 (−0.77, 0.78)
0.12 (−1.18, 1.42)	MR	0.25 (−1.19, 1.69)	0.12 (−0.91, 1.16)
−0.13 (−1.40, 1.13)	−0.25 (−1.69, 1.19)	ING	−0.13 (−1.12, 0.87)
−0.00 (−0.78, 0.77)	−0.12 (−1.16, 0.91)	0.13 (−0.87, 1.12)	Control

### 3.8 Meta-regression analysis

**Figure 12:**
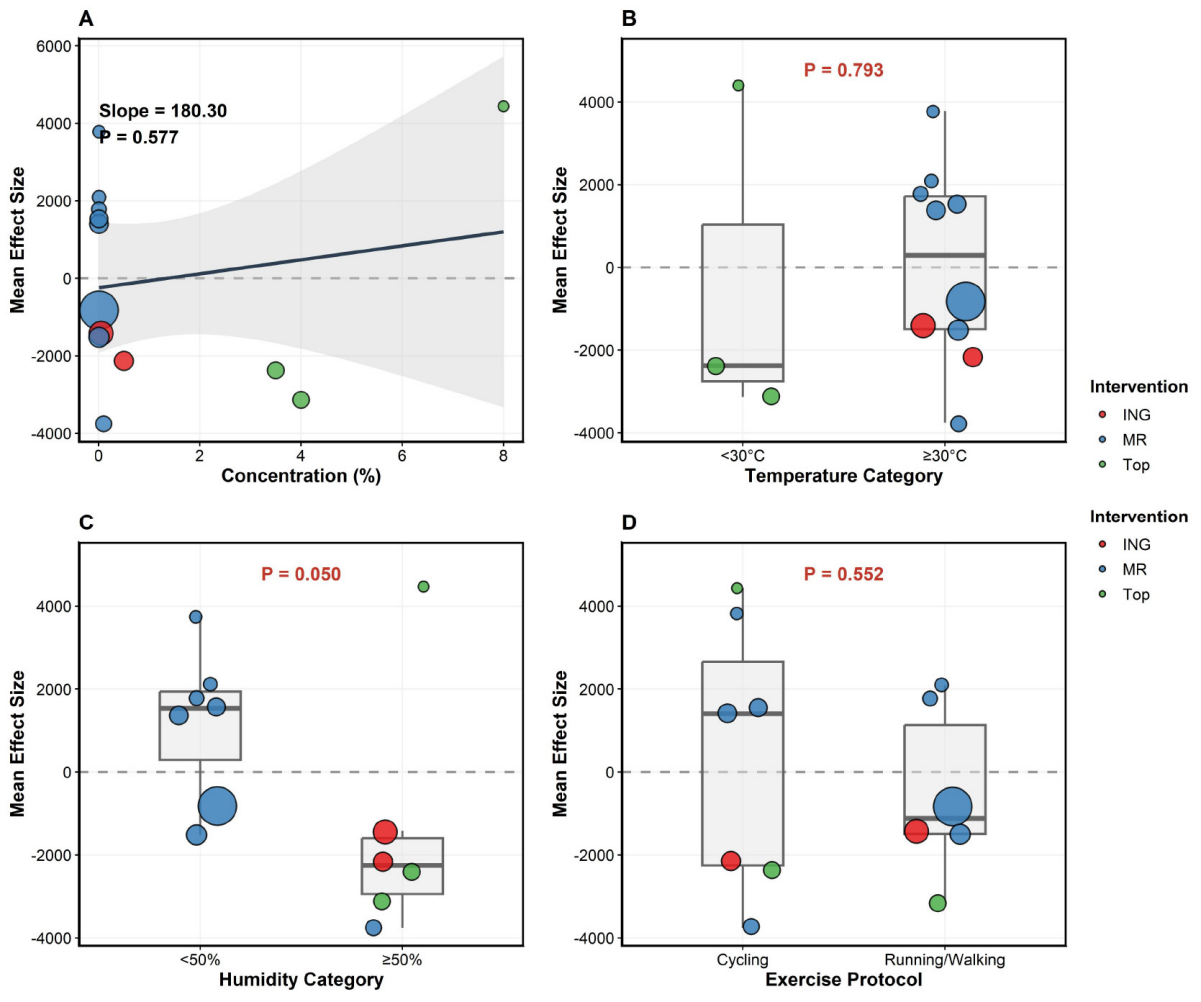
Moderator analyses exploring the effects of potential moderators on the ergogenic efficacy of menthol in exercise endurance. **(A)** Meta-regression of menthol concentration; circle size indicates study weight. **(B–D)** Subgroup analyses by ambient temperature **(B)**, relative humidity [**(C)**; *P* = 0.050], and exercise protocol **(D)**. Box plots represent the median, IQR, and range.

To investigate potential sources of heterogeneity, moderator analyses—including both meta-regression and subgroup analyses—were performed across all outcomes. Notably, for exercise endurance, relative humidity emerged as a critical moderator (*P* = 0.050; Figure 12), suggesting that the ergogenic efficacy of menthol is significantly attenuated in high-humidity environments (≥ 50%). Specifically, in low-humidity conditions (< 50%), where all included trials utilized the mouth rinsing modality (*n* = 7), menthol exhibited a robust ergogenic effect. Conversely, the high-humidity subgroup (≥ 50%)—which comprised a mix of MR (*n* = 1), ingestion, and topical applications—demonstrated a markedly diminished overall efficacy. We systematically attempted to further explore this humidity effect through subgroup analyses. However, a full network subgroup analysis was precluded because the exclusive use of MR in the low-humidity condition resulted in a disconnected network structure. A subsequent attempt to perform a direct pairwise comparison for the MR modality across the two humidity levels was also unfeasible due to extreme data sparsity (7 trials vs. 1 trial).

By contrast, no significant moderating effects were observed for factors such as menthol concentration, ambient temperature, or exercise protocols regarding the other three outcome measures: core temperature, heart rate, and mean power output (*P* > 0.05; see Supplementary Figures S2–S4). The lack of statistical significance in these findings may be attributed to the relatively small number of primary studies and limited sample sizes, which potentially constrained the statistical power to detect subtle moderating influences.

### 3.9 Sensitivity analysis for result robustness

The robustness of the primary outcomes was further verified through a leave-one-out sensitivity analysis (Supplementary Figure S1) (47). The results remained highly stable across all iterations; specifically, the exclusion of any individual study did not fundamentally alter the direction or the statistical significance of the pooled effect sizes for exercise endurance, core temperature, heart rate, or mean power. This indicates that the current findings are not driven by any single influential study.

### 3.10 Publication bias

**Figure 13:**
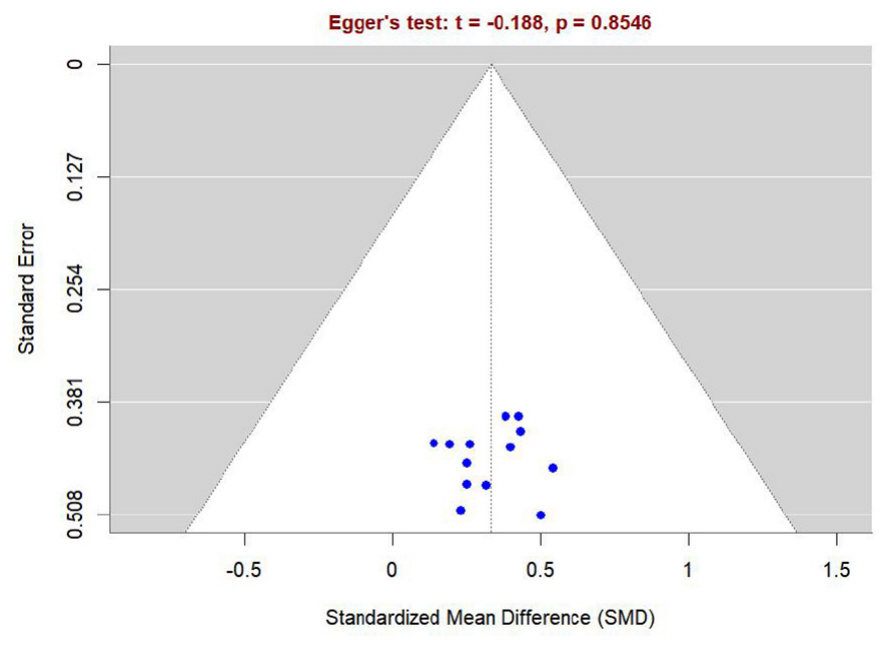
Funnel plot with Egger's test for endurance performance.

Publication bias for endurance performance indicators was assessed using an Egger funnel plot (see Figure 13). Although the funnel plot exhibited a degree of visual asymmetry, the *P*-value from Egger's linear regression test was 0.8546 (exceeding the significance threshold of 0.05). This indicates that the pooled results for endurance performance are relatively robust, with no significant risk of publication bias detected. For outcomes including mean power output, core temperature, and heart rate, Egger's test was not performed because the number of included studies was fewer than ten (*n* < 10). In such cases, the statistical power of the test is insufficient to reliably detect asymmetry in the funnel plot (48).

### 3.11 Certainty of evidence

The certainty of evidence for the outcomes included in this systematic review ranged from “very low” to “low.” Specifically, the quality of evidence for endurance performance was graded as low (12 studies, *n* = 272). While the Surface Under the Cumulative Ranking (SUCRA) analysis suggested a potential hierarchical advantage for the ingestion route, the strength of this inference is limited. Consequently, this outcome was downgraded due to concerns regarding imprecision, as evidenced by the 95% confidence intervals crossing the null point and the relatively modest total sample size (Downgrade c).

The certainty of evidence for the two core performance metrics—mean power (4 studies, *n* = 72) and heart rate (4 studies, *n* = 70)—was assessed as very low. Both indicators were downgraded due to a high risk of bias arising from blinding challenges and serious imprecision; the latter was characterized by a limited number of participants and extremely wide 95% CIs that substantially overlapped the null value (Downgrades a, d).

The evidence quality for physiological response indicators, such as core temperature (5 studies, *n* = 86), was also categorized as very low. SUCRA rankings revealed a high degree of consistency between various menthol interventions and the control group, potentially reflecting a “decoupling” phenomenon between performance and physiological load. This outcome was downgraded primarily due to serious imprecision stemming from negligible effect sizes and a lack of statistical significance (Downgrades a, d).

Regarding the comparative efficacy of administration routes, the evidence for topical application in core temperature regulation was of very low quality. This was evidenced by a notably low SUCRA value (6.2%) and the least favorable MeanRank. This metric was further compromised by imprecision related to sample size scarcity and potential heterogeneity, as the intervention medium may have incidentally hindered evaporative heat dissipation (Downgrades a, d).

Notably, with the exception of endurance performance, all other outcomes—including mean power, core temperature, and heart rate—involved fewer than 10 studies, which precluded the application of Egger's test for a robust assessment of publication bias. Furthermore, the unique physicochemical properties of menthol meant that most included studies carried “some concerns” regarding bias in the blinding process, which may subtly influence the overall certainty of the findings. Given that the certainty of evidence for most outcomes was rated as “low” to “very low,” the conclusions regarding the ergogenic efficacy of menthol in the heat should be interpreted with significant caution (Table 12).

**Table 12:** GRADE evidence quality evaluation.

Outcome	No. of studies (Participants)	Risk of bias	Inconsistency	Indirectness	Imprecision	Publication bias	Quality of evidence
Endurance performance	12 (272)	Downgraded a	Not downgraded	Not downgraded	Downgraded c	No significant bias e	⊕⊕○○ Low
Mean power output	4 (72)	Downgraded a	Not downgraded	Not downgraded	Seriously downgraded d	Not evaluated f	⊕○○○ Very low
Core temperature	5 (86)	Downgraded a	Not downgraded	Not downgraded	Seriously downgraded d	Not evaluated f	⊕○○○ Very low
Heart rate	4 (70)	Downgraded a	Not downgraded	Not downgraded	Seriously downgraded d	Not evaluated f	⊕○○○ Very low

## 4 Discussion

### 4.1 Summary of findings

This network meta-analysis evaluated the relative efficacy of ingestion, mouth rinsing, and topical application on exercise performance and physiological responses in the heat, suggesting that menthol's ergogenic benefits may follow distinct task-specific trends. SUCRA results indicated that ING had the highest potential probability (76.1%) for prolonging exercise endurance, while MR demonstrated a superior tendency for enhancing mean power output (66.9%); however, the impact on core temperature (Tc) and heart rate (HR) appeared relatively modest across all administration routes. These SUCRA results should be interpreted as exploratory trends rather than definitive evidence, as pairwise comparisons revealed that most 95% confidence intervals (CIs) crossed the null point, which might be attributed to the limited statistical power resulting from the small sample sizes (*n* = 8–22) typical of exercise physiology research. Environmental factors may further influence these outcomes, with meta-regression identifying relative humidity as a potential moderator (*P* = 0.050); specifically, the ergogenic effect appeared robust in low-humidity conditions (< 50%) but was noticeably attenuated in high-humidity environments (≥ 50%), possibly because the potential risk of impaired evaporative cooling might have offset the perceived cooling benefits. Furthermore, leave-one-out sensitivity analysis supported the overall stability of these findings across all primary outcome measures, indicating that the results were not driven by any single influential study. Overall, the ergogenic efficacy of menthol in the heat appears to be a perception-driven process potentially modulated by both the specific exercise task and environmental humidity, providing a probabilistic evidence-based framework for personalized supplementation strategies in athletic settings.

### 4.2 Comparison with previous studies and mechanism analysis

The network meta-analysis (NMA) results of the present study are generally consistent with previous pairwise meta-analyses while further extending the literature by comparing multiple menthol administration routes within a unified analytical framework. Overall, mucosal or internal interventions—represented by mouth rinsing and ingestion—appear to demonstrate more stable positive trends in enhancing endurance performance and modulating thermal perception under heat stress. This finding aligns with earlier evidence suggesting that periodic menthol rinsing may blunt subjective thermal sensations and bolster exercise output (35, 45, 49). At the same time, the present findings should be interpreted cautiously, because most pairwise comparisons remained non-significant and the corresponding confidence intervals crossed the null value; therefore, the current data support probabilistic ranking trends rather than definitive evidence of route superiority (15, 50). This more conservative interpretation is also consistent with recent evidence showing that menthol mouth rinsing does not significantly improve exercise capacity or performance overall at the group level, although endurance-type tasks may still derive context-specific benefit (50). Likewise, the earlier meta-analysis by Jeffries et al. reported a modest overall ergogenic effect of menthol and suggested relatively greater benefits for internal application, which is broadly concordant with the present ranking pattern favoring ING for endurance-related outcomes (15). Our analysis further nuances this perspective: ING may offer a potential advantage in sustaining long-term endurance (e.g., TT or TTE), whereas MR exhibits a greater inclination toward improving immediate mean power output. In contrast, the effects of topical application are characterized by notable heterogeneity; although it might briefly improve thermal comfort, its actual contribution to performance enhancement remains debatable (34), which is consistent with the relatively lower ranking of the Top modality observed in our NMA. When considered together with the humidity-dependent pattern identified in the meta-regression, these route-related differences appear more likely to be context-sensitive than universally generalizable, especially under hot-humid conditions in which evaporative heat loss is already constrained.

The route-specific selectivity observed in this study could potentially be explained by the distinct neurophysiological mechanisms they trigger. As an agonist of cold-sensitive receptors such as TRPM8, menthol is thought to enhance performance primarily by transmitting non-thermal “cooling” signals to the central nervous system, thereby “tricking” the brain's thermoregulatory center (51). This mechanism likely contributes to the “decoupling” state observed between performance gains and objective physiological markers—such as core temperature and heart rate—whereby participants maintain higher exercise output and motivation by blunting their subjective perception of thermal strain, even in the absence of substantive physiological cooling (14, 52). This decoupling phenomenon can be interpreted through two complementary theoretical lenses—the Central Governor Model and the Psychobiological Model of endurance performance (16, 53). From the perspective of the Central Governor Model, menthol-induced cooling cues may reduce the salience of afferent thermal threat signals and thereby delay centrally mediated downregulation of motor output, allowing athletes to maintain pace or work rate despite persistent physiological heat strain (53). From the perspective of the Psychobiological Model, menthol may lower perceived effort and thermal discomfort without materially changing physiological load, thereby altering conscious pacing decisions and increasing tolerance for sustained effort; in this framework, improved performance is mediated more by perception and motivation than by genuine heat dissipation (16, 18). Because the present NMA found only modest effects on core temperature and heart rate, the current evidence more strongly supports a perceptual-regulatory mechanism than a true thermolytic mechanism (15, 22).

These neuro-perception-based differences may further elucidate the specific advantages of different administration routes; specifically, the transient stimulation of the oral mucosa during mouth rinsing may rapidly activate oral cold receptors and trigeminal afferent pathways, generating an immediate cooling percept that may be particularly relevant for short-duration or power-oriented tasks (22, 41, 54). This interpretation is also compatible with the recent MR-specific meta-analysis, which failed to show a significant overall pooled effect yet still suggested that endurance exercise may be the setting in which this strategy is most likely to confer benefit (50). In contrast, the ingestion process potentially provides sustained activation of internal receptors in the esophagus and gastrointestinal tract, creating an “inside-out” and more persistent cooling sensation that may be better suited for the demands of long-duration endurance exercise (55, 56). However, this apparent advantage should still be regarded as hypothesis-generating rather than conclusive, because direct head-to-head mechanistic comparisons between ING and MR remain scarce (15, 22). Furthermore, our analysis of topical application provides a basis for physiological reflection on its application boundaries, as the observation that Top performed poorly—or even showed a slight negative trend—in core temperature regulation might involve a “cross-inhibition” effect in the thermoregulatory center where the body misinterprets the external environment as cooler than it is, thereby prematurely reducing sweat rate or inducing peripheral vasoconstriction (57), while the gel or spray medium might also act as a minor physical barrier to sweat evaporation (58). This interpretation is supported by experimental evidence indicating that topical menthol can delay sweating, alter skin blood flow, and in some conditions impair whole-body thermoregulation despite making the environment feel cooler (23, 57). Accordingly, the practical value of menthol in the heat should be framed in moderate terms: menthol may be useful as a perception-oriented adjunct under specific task and environmental conditions, but it should not be interpreted as a substitute for physiological cooling, nor as evidence that thermal risk has been reduced (22). In summary, the ergogenic effect of menthol in the heat appears to be essentially a “perception-driven” neuromodulatory process, and because the present study did not directly measure physiological or neural pathway indicators, these inferences remain primarily based on existing theoretical frameworks, suggesting that future research is warranted to incorporate direct monitoring to further verify how menthol balances the conflict between improved subjective perception and objective thermoregulation.

### 4.3 Practical implications

Based on the exploratory trends and the overall low-to-very-low certainty of evidence from this network meta-analysis, practical recommendations for menthol supplementation must be approached with caution. While the results indicate probabilistic trends rather than definitive statistical superiority, practitioners might consider a task-oriented approach: ingestion could serve as a potential adjunct for prolonged endurance events, whereas mouth rinsing might be tentatively prioritized for tasks demanding instantaneous power output. Furthermore, these strategies should be highly contextualized to ambient humidity, as menthol's ergogenic potential appears largely restricted to low-humidity environments (< 50% RH) and noticeably attenuates in high-humidity conditions where natural evaporative heat loss is already compromised. Crucially, safety remains the paramount concern; because menthol primarily blunts the subjective perception of thermal stress without meaningfully reducing objective physiological strain, it creates a “decoupling” effect that could allow athletes to inadvertently push beyond safe hyperthermic limits. Therefore, menthol must never substitute for genuine physical cooling interventions and should strictly be paired with objective physiological monitoring to ensure athlete safety. Finally, topical application is currently not recommended as a reliable ergogenic aid, as it consistently demonstrated the weakest potential for performance and thermoregulation in this analysis and may even act as a physical barrier to sweat evaporation.

### 4.4 Study limitations

While this study utilizes a Network Meta-Analysis (NMA) framework to integrate findings across various administration routes, several limitations should be noted. First, the evidence base is constrained by relatively small sample sizes (totaling approximately 165 participants, with individual trials typically involving 8–22 individuals) and limited statistical power. This increases the risk of Type II errors and results in wide confidence intervals, particularly for outcome networks like mean power output and heart rate that are based on only four studies. Consequently, the lack of statistical significance in most pairwise comparisons means the SUCRA rankings should be interpreted as exploratory trends rather than definitive evidence of superiority. Second, the NMA features a sparse, star-shaped network structure lacking closed loops; because comparisons between active menthol routes are entirely indirect via a common control, the robustness of consistency assessments and the stability of hierarchical rankings may be diminished. Third, there is potential confounding between specific environmental moderators and intervention modalities, as subgroup categories for temperature and humidity perfectly overlapped with delivery methods in certain physiological networks. For instance, trials at < 30 °C exclusively used topical application, while those at ≥30 °C predominantly used mouth rinsing or ingestion, Similarly, regarding relative humidity, our systematic attempts to isolate its effect on exercise endurance via network subgroup analyses or direct pairwise comparisons for MR were precluded by disconnected network structures and extreme data sparsity (i.e., 7 MR trials in low-humidity vs. only 1 in high-humidity conditions). These overarching structural constraints make it difficult to isolate environmental effects from the physiological impacts of the delivery methods themselves. Finally, the unique sensory properties of menthol rendered true double-blinding practically unattainable, introducing a potential risk of bias for perception-related outcomes. Future large-scale, multi-arm randomized controlled trials utilizing rigorous placebo designs are warranted to enhance statistical power, decouple environmental factors from administration strategies, and provide a more robust evidence base for personalized menthol supplementation.

## 5 Conclusion

This systematic review and network meta-analysis provides an integrated evaluation of the relative efficacy of three menthol administration routes—ingestion, mouth rinsing, and topical application—on exercise performance and physiological responses in the heat. The findings suggest that active menthol application may exhibit a positive trend in supporting exercise performance under heat stress. While SUCRA probabilities point toward potential outcome-specific trends—such as ING for prolonging endurance performance and MR for enhancing mean power output—these rankings must be explicitly interpreted as exploratory. Given that the majority of pairwise comparisons did not reach statistical significance, with 95% confidence intervals consistently crossing the null point, these SUCRA-based hierarchies are indicative rather than a definitive demonstration of route superiority. Consistent with existing literature, a distinct “decoupling” was observed between potential performance gains and objective physiological responses. The intervention routes showed no statistically significant differences in regulating core temperature or heart rate compared to control conditions, suggesting that any ergogenic effects of menthol likely stem from the alteration of subjective thermal perception rather than substantive physical cooling. Consequently, practical interpretations regarding a task-oriented supplementation approach should be framed cautiously as preliminary hypotheses. The current evidence base is inherently limited by insufficient statistical power derived from small-sample primary studies, strictly requiring further high-quality, large-scale randomized controlled trials to substantiate these exploratory trends. Finally, because menthol possesses the potential to mask actual thermal stress without alleviating physiological heat strain, any application of these strategies in practice must be accompanied by objective physiological monitoring to ensure athlete safety.

## Data Availability

The original contributions presented in the study are included in the article/supplementary material, further inquiries can be directed to the corresponding author.
