# Supplementary material for: Effects of different menthol administration routes on endurance performance and physiological responses in the heat: a network meta-analysis
Source: Front Nutr. 2026 May 18;13:1833420. doi: 10.3389/fnut.2026.1833420 (PMC13223170; doi:10.3389/fnut.2026.1833420)
Supplement: Supplementary file 1 [file Supplementary_File_1.docx]

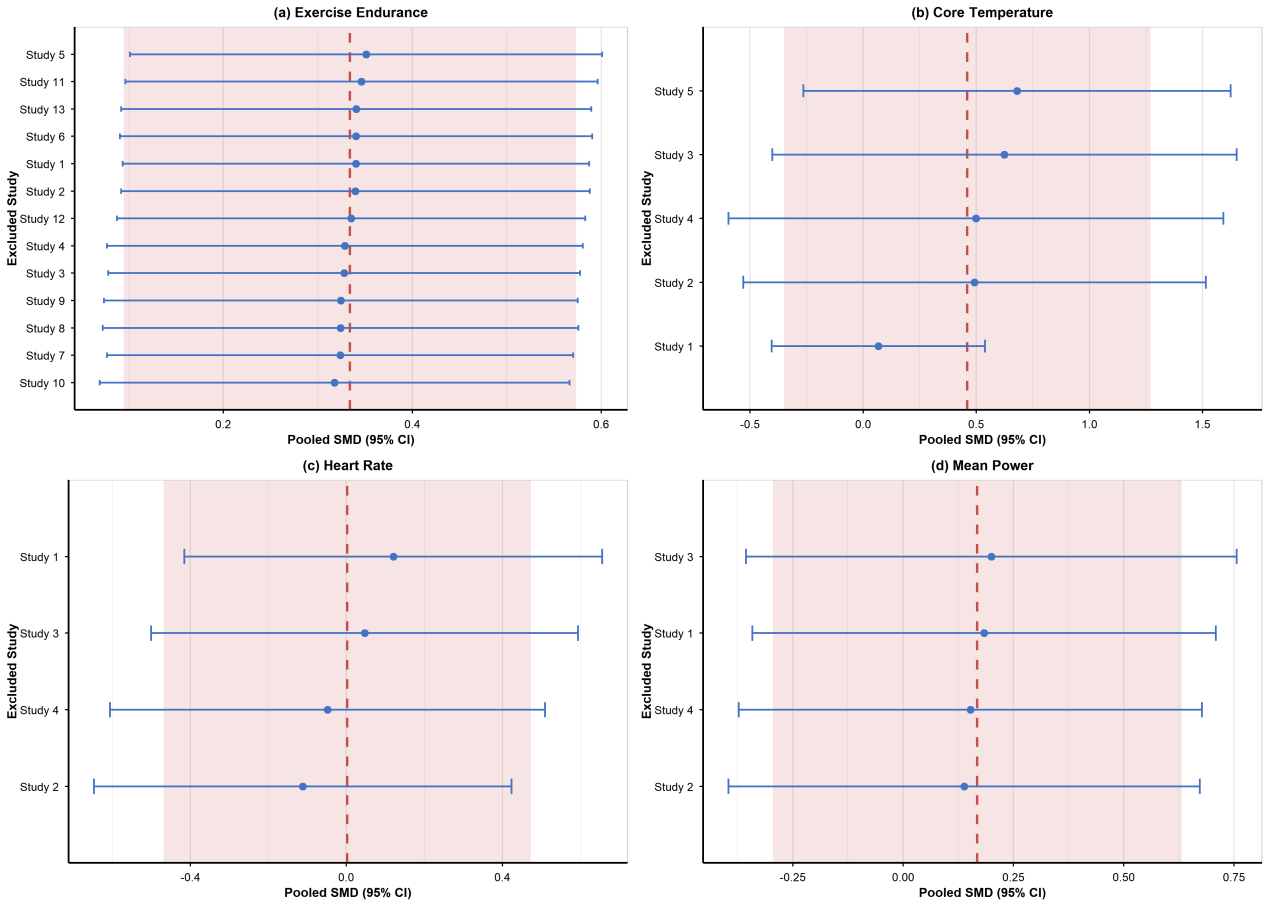


Supplementary Figure S1 Leave-one-out sensitivity analysis for primary outcomes.

Note: Each plot represents the re-calculated pooled standardized mean difference (SMD) and its 95% confidence interval after excluding the corresponding study listed on the Y-axis. The vertical dashed line indicates the original overall effect size. (a) Exercise Endurance; (b) Core Temperature; (c) Heart Rate; (d) Mean Power.


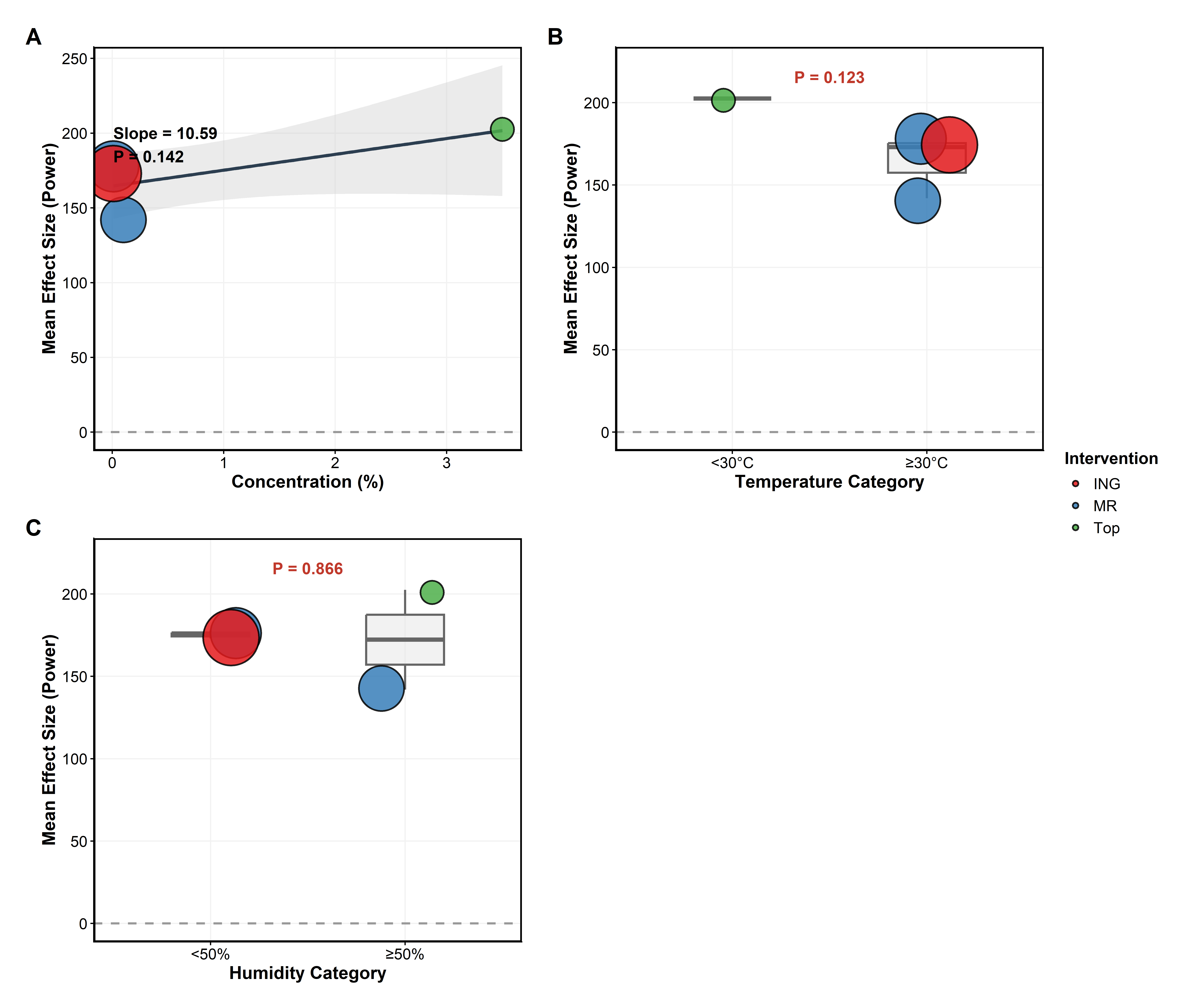


Supplementary Figure S2 Moderator analyses exploring the effects of potential moderators on the ergogenic efficacy of menthol in average power

Moderator analyses indicate that the ergogenic efficacy of menthol on average power is not significantly influenced by concentration, temperature, or humidity. Specifically, meta-regression reveals a slight but non-significant positive relationship between menthol concentration and effect size (Slope = 10.59, P = 0.142). Subgroup analyses further demonstrate no statistically significant differences in performance outcomes across different environmental conditions, including temperature (<30°C vs. ≥30°C; P = 0.123) and humidity (<50% vs. ≥50%; P = 0.866). Overall, these findings suggest that the benefits of menthol interventions on power output remain relatively consistent and are independent of the dosage concentration or variations in environmental heat and moisture.


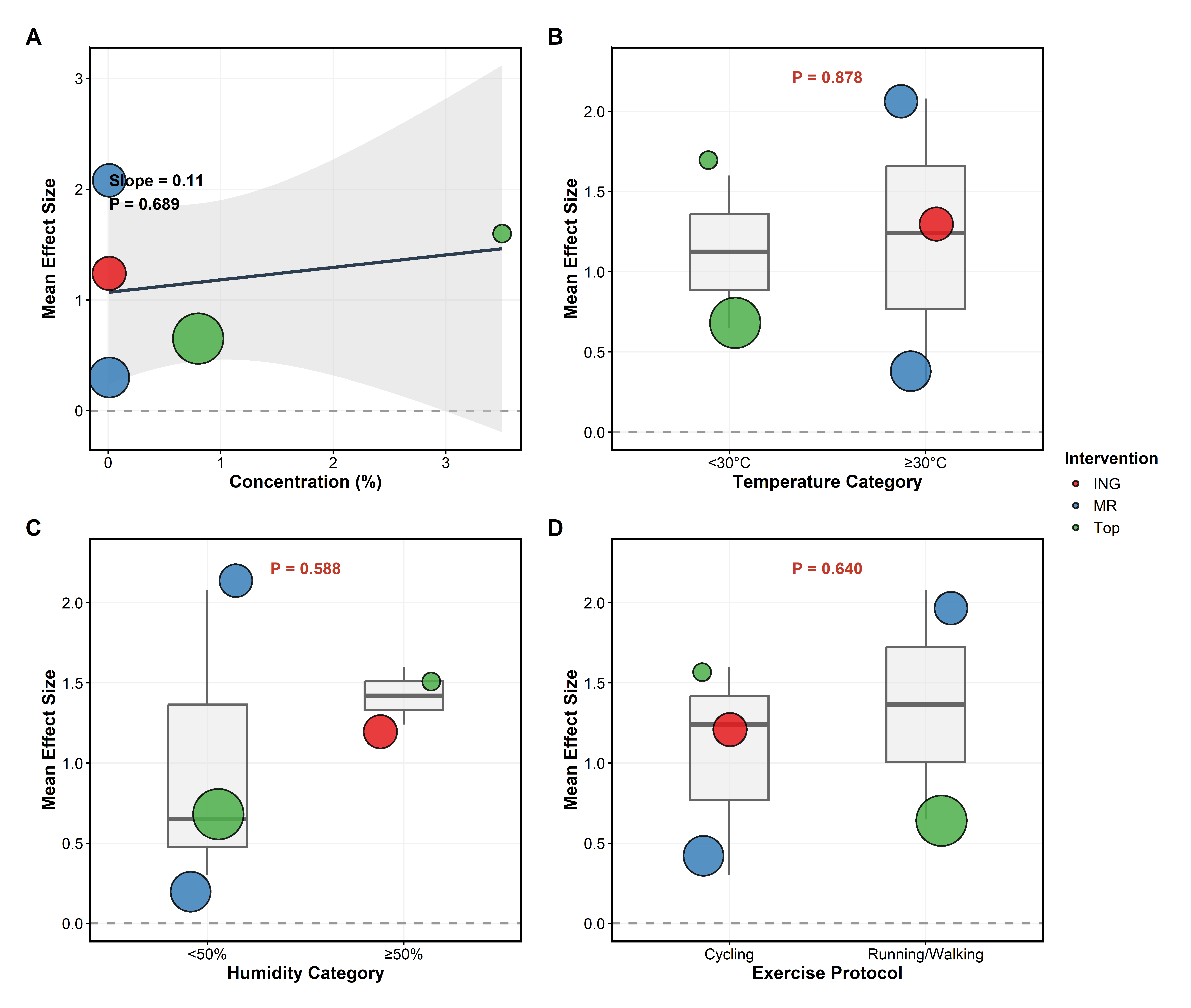


Supplementary Figure S3 Moderator analyses exploring the effects of potential moderators on the efficacy of menthol in core temperature

Moderator analyses indicate that the effect of menthol interventions on core temperature is not significantly influenced by concentration, temperature, humidity, or exercise protocol. Specifically, meta-regression shows a negligible and non-significant relationship between menthol concentration and effect size (Slope = 0.11, P = 0.689, Panel A). Furthermore, subgroup analyses reveal no statistically significant differences across different environmental conditions, including temperature (<30°C vs. ≥30°C; P = 0.878, Panel B) and humidity (<50% vs. ≥50%; P = 0.588, Panel C), as well as exercise modalities (Cycling vs. Running/Walking; P = 0.640, Panel D). Overall, these findings suggest that the impact of menthol on core temperature remains relatively consistent regardless of dosage, environmental heat and moisture, or the type of exercise performed.


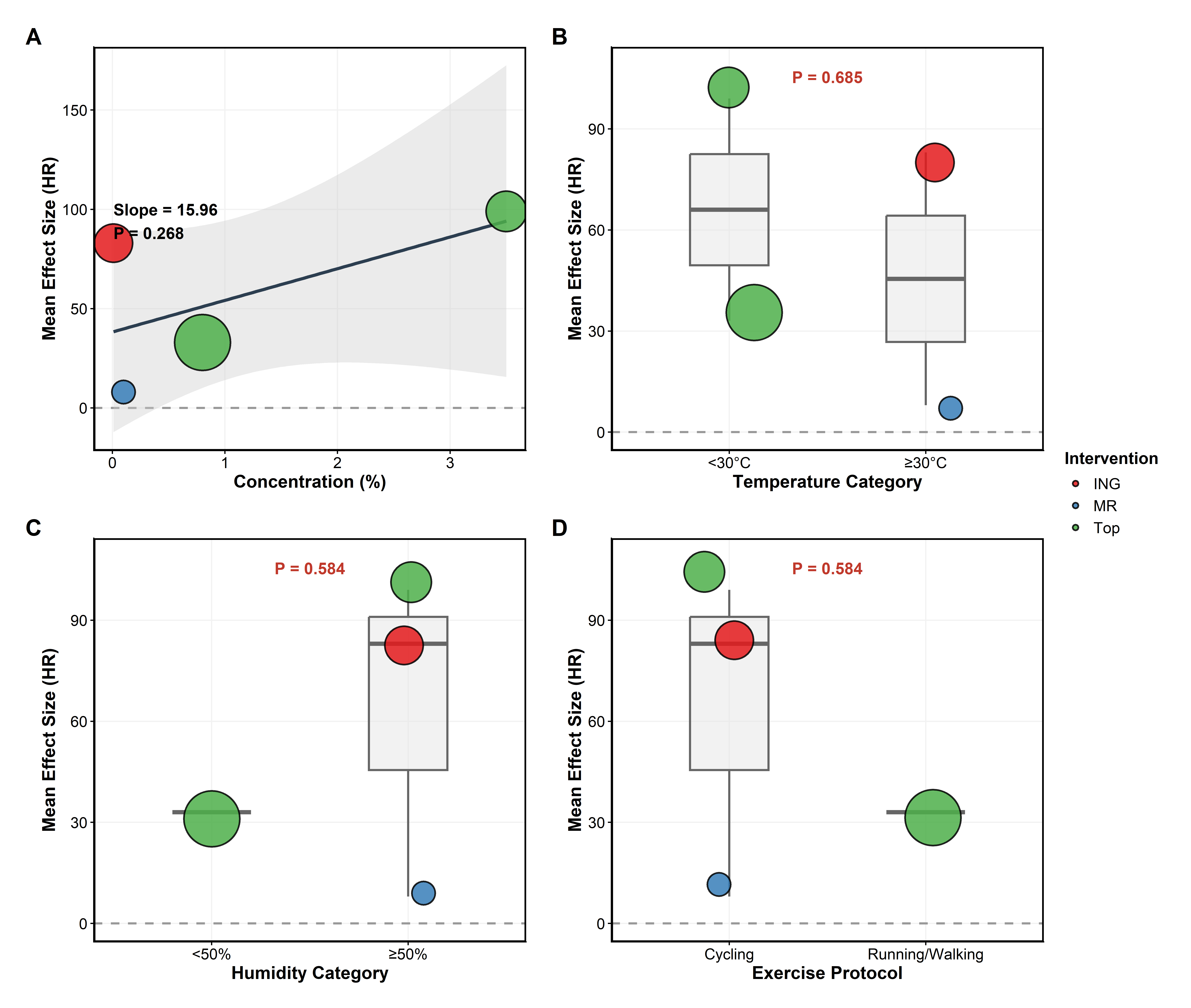


Supplementary Figure S4 Moderator analyses exploring the effects of potential moderators on the efficacy of menthol in heart rate

Moderator analyses indicate that the effect of menthol interventions on heart rate (HR) is not significantly influenced by concentration, temperature, humidity, or exercise protocol. Specifically, meta-regression reveals a non-significant positive relationship between menthol concentration and effect size (Slope = 15.96, P = 0.268, Panel A). Furthermore, subgroup analyses demonstrate no statistically significant differences across different environmental conditions, including temperature (<30°C vs. ≥30°C; P = 0.685, Panel B) and humidity (<50% vs. ≥50%; P = 0.584, Panel C), or between exercise modalities (Cycling vs. Running/Walking; P = 0.584, Panel D). Overall, these findings suggest that the impact of menthol on heart rate remains relatively consistent and is independent of the intervention dosage, environmental heat and moisture, or the specific exercise type.
